# The impact of extreme air pollution on preterm birth in twin pregnancies: identifying susceptible exposure windows

**DOI:** 10.1080/07853890.2025.2534854

**Published:** 2025-07-20

**Authors:** Wei-Ze Xu, Wei-Zhen Tang, Qin-Yu Cai, Yun-Ren Pan, Ying-Xiong Wang, Ya Chen, Xia Lan, Hong-Yu Xu, Zi-Heng Zhang, Bo-Yuan Deng, Li Wen, Lan Wang, Tai-Hang Liu

**Affiliations:** a Department of Obstetrics and Gynecology, Women and Children’s Hospital of Chongqing Medical University, Chongqing, China; b Department of Bioinformatics, School of Basic Medical Sciences, Chongqing Medical University, Chongqing, China; c The Joint International Research Laboratory of Reproduction and Development, Chongqing Medical University, Chongqing, China

**Keywords:** Twin pregnancy, air pollution, preterm birth, distributed lag model, susceptible exposure window, cumulative effect

## Abstract

**Background:**

Accelerated industrialization globally has intensified air pollution, but the susceptibility periods for extreme air pollution in twin pregnancies remain undefined.

**Methods:**

This study investigated the association between extreme air pollution exposure and preterm birth risk in twin pregnancies. Data on 3623 twin pregnancies in Chongqing from 2017 to 2022 and air pollution readings from 12 monitoring stations were analyzed using distributed lag non-linear quasi-Poisson regression models. Additionally, four extreme air pollution indices were developed to assess the cumulative effects of lagged exposures on preterm birth risk through multivariate logistic regression.

**Results:**

Compared to the lower quartile, the 95th percentile of extreme air pollution exposure showed a positive correlation between concentrations of PM_2.5_, PM_10_, NO_2_, SO_2_ and CO and preterm birth risk in twin pregnancies, with O_3_ inversely correlated. Sensitive periods for air pollutants were different. 8–12 and 27–35 gestational weeks were identified for PM_2.5_; 6–13 and 27–35 gestational weeks were identified for PM_10_; 5–14 and 21–33 gestational weeks were identified for NO_2_; 4–15 and 24–36 gestational weeks were identified for SO_2_; 4–11 and 29–33 gestational weeks were identified for CO. PM_2.5_, PM_10_, SO_2_ and O_3_ showed cumulative effects across short and long lags, while CO showed a long-term effect. Notably, NO_2_ exhibited a protective effect during all lag periods.

**Conclusion:**

The study highlights gestational windows of 8–11 and 29–33 weeks as highly sensitive to extreme pollution for preterm birth in twin pregnancies, with marked risk increases during 0–3, 0–6 and 0–9-month lag periods.

## Introduction

Air pollution has become a formidable global health hazard, with an estimated 91% of the global population living in areas with air quality below World Health Organization standards, contributing to roughly 4.2 million fatalities each year. Alarmingly, a disproportionate 89% of these deaths transpire in low- and middle-income nations [1]. The relentless march of industrialization has escalated the severity and regularity of extreme pollution events, broadening their impact to encompass a wider geographical scope and thereby exacerbating the threat to public health. The harmful effects of air pollution affect all populations, while pregnant women are particularly vulnerable due to their unique physio­logical conditions. Preterm birth (PTB), defined as childbirth occurring before 37 weeks of gestation, is reported in 5% to 18% of births across 184 countries (WHO). PTB stands as a principal cause of neonatal morbidity and mortality [2] and is linked to enduring physical, cognitive and developmental challenges (WHO), intensifying the strain on families and straining healthcare, education and social services [3]. The mechanisms underlying preterm birth are complex and have not yet been fully elucidated [4]. Existing studies have shown that the initiation of labour is closely related to a variety of biological processes, especially the migration of leukocytes to the uterus, the release of chemokines and cytokines and the activation of inflammatory signalling pathways [5]. Abnormal initiation of these physiological processes may be associated with a range of pathological factors, such as infection, cervical incompetence, allergic reactions, psychological stress, uterine overdistension and uterine ischaemia [6]. In recent years, environmental exposure, particularly air pollution during pregnancy, has gradually become a focus of research on its potential impact on preterm birth. In twin pregnancies, physiological factors such as larger placentas, larger foetal volumes and increased amniotic fluid volume may exacerbate the impact of air pollution on pregnancy [7]. Due to the unique physiological burden of twin pregnancies, these pregnant women may be more sensitive to long-term exposure to air pollutants. Systemic inflammatory responses, oxidative stress and hemodynamic changes caused by air pollution may be more pronounced in this population [8,9]. However, research on twin pregnancies remains relatively scarce.

The prevalence of twin pregnancies has surged alongside the widespread adoption of assisted reproductive technologies, now accounting for 3% of all live births and approximately 15–20% of preterm births [10,11]. Twin pregnancies face more complex physiological challenges compared with singleton pregnancies, particularly in terms of placental function and uterine burden. Twin pregnancies are often associated with abnormal placental blood supply and higher uterine tension, which increases the risk of placental dysfunction [12]. Moreover, compared with singleton pregnancies, the risk of pregnancy complications in twin pregnancies is approximately five times higher, significantly heightening the likelihood of adverse maternal outcomes such as preeclampsia, gestational diabetes and caesarean delivery [13], as well as foetal complications like growth restriction, preterm delivery and mortality [14]. Notably, PTB rates in twin pregnancies stand at 65.1% [15], with neurological disorder risks in preterm twins at 15.4%, substantially surpassing the 4.1% in preterm singletons [16,17]. Investigating air pollutant exposure during twin gestations is crucial for pinpointing air pollution thresholds and identifying gestational sensitive windows, which are essential for developing prophylactic strategies and guiding pregnant women on when to implement preventive measures against specific pollution levels or during particular gestational weeks. Therefore, exploring the impact of air pollution on the risk of preterm birth in twin pregnancies holds significant clinical and research value.

Past studies have endeavoured to pinpoint sensitive windows within the gestational period that are particularly susceptible to air pollution’s adverse effects [18–20], aiming to inform prenatal care practices, such as behavioural strategies to mitigate exposure [19,20]. Research often targets the first trimester, the entirety of gestation, or specific months, yet findings are inconsistent [21–23]. The biological response of pregnant women to air pollution may not align with gestational stages, suggesting that correlations with specific gestational times may not sufficiently identify sensitive windows. Given the dynamic nature of air pollution exposure, which can lead to time-dependent, cumulative and delayed reproductive health effects [24,25], distributed lag models (DLMs) are apt for modelling risks based on past exposure intensity and timing, thus suited for detecting susceptible windows [26,27]. DLMs have been employed in studies examining singleton PTB to identify the most vulnerable periods to air pollution, indicating that mid to late gestation may be particularly susceptible [26,28]. Nonetheless, research on sensitive exposure windows for PTB in twin pregnancies is scant, particularly in light of the distinct physiological characteristics between singleton and twin gestations.

In response to the ongoing debate regarding the definition of extreme air pollution exposure, our study adopts a novel approach by converting average pollution concentration exposure to the frequency of surpassing extreme pollution thresholds. This method has been substantiated in an extensive study involving over 70,000 singleton pregnancies [29]. Utilizing (distributed lag non-linear model) DLNM, we aim to thoroughly examine the lag-dose-response relationship between air pollutant concentrations and PTB risk in twin pregnancies, emphasizing the cumulative effects of extreme air pollution exposure on PTB risk in twin gestations.

## Materials and methods

### Data collection

All data were obtained from the electronic medical records (EMR) database at the Women’s and Children’s Hospital of Chongqing Medical University, encompassing basic details about the pregnant women as well as pregnancy-related information.

## Study design

### Study participants

**Figure 1.**
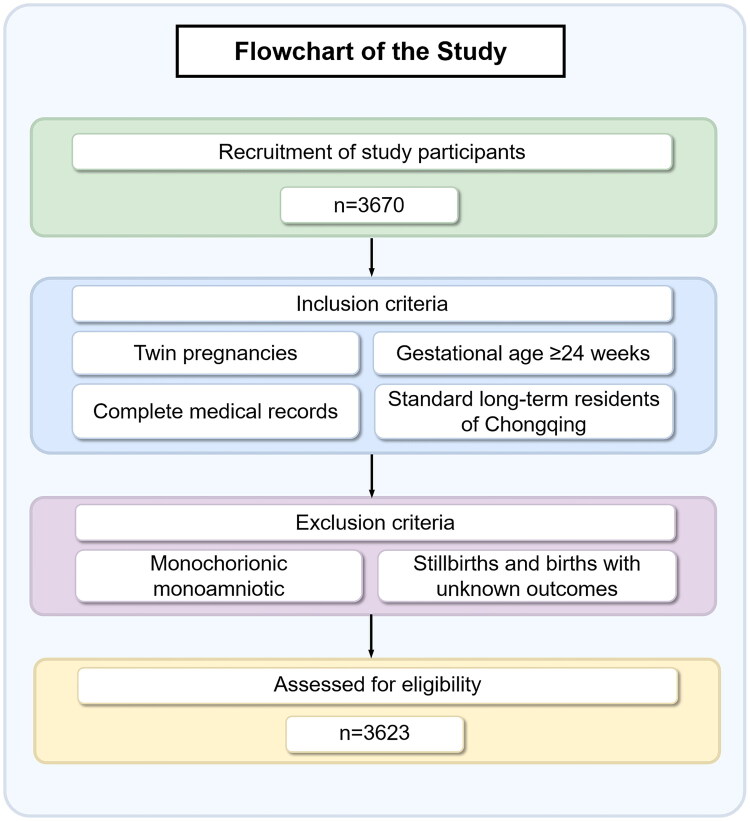
Flowchart of this retrospective cohort study.

This retrospective cohort study was conducted at the Women’s and Children’s Hospital of Chongqing Medical University in China. The study retrospectively included twin pregnancies in women who underwent regular prenatal check-ups and gave birth between January 2017 and December 2022. The inclusion criteria were as follows: (i) gestational age ≥24 weeks; (ii) complete medical records; (iii) twin pregnancy; (iv) long-term residence in Chongqing city without any plans to relocate during pregnancy; (v) Professionals conducted an initial screening based on participants’ residence distance from the hospital to ensure accurate pollution exposure assessment. The exclusion criteria were: (i) monoamniotic twin pregnancies; (ii) stillbirths and births with unclear outcomes. A total of 3670 pregnant women met the inclusion criteria. After applying the exclusion criteria, 3623 cases were ultimately included in the study. The flowchart of the study inclusion and exclusion process is shown in Figure 1.

### Air pollution exposure assessment

Located in the southwestern part of China, Chongqing covers a total area of 82,400 square kilometres, approximately 75% of which is mountainous terrain. As of the end of 2021, the city is composed of 38 districts and counties, with a total population reaching 31.2 million. Chongqing primarily consists of four regions: the central area, the western area, the northeastern area and the southeastern area. The central area features relatively flat terrain, a more developed economy and a higher degree of urbanization. In contrast, the other regions are predominantly mountainous, with slower economic development and a scarcity of medical resources.

**Figure 2.**
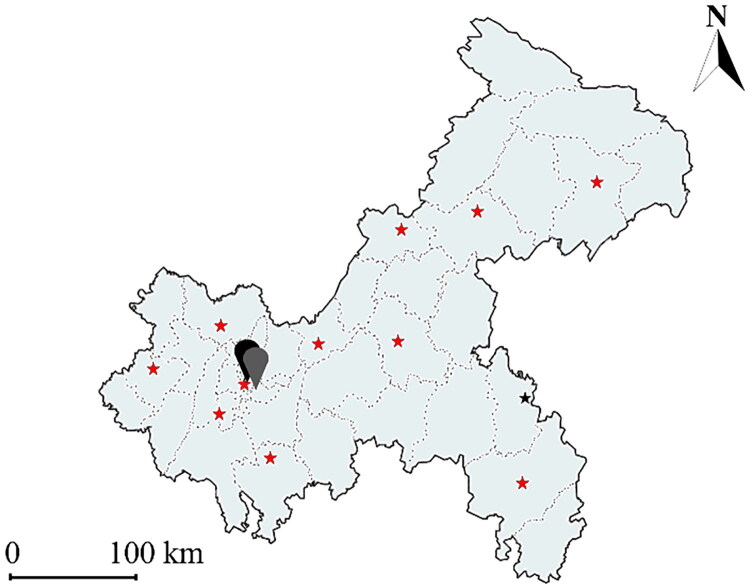
The geographical distribution map of meteorological monitoring stations in Chongqing, China.

Chongqing is equipped with 12 basic meteorological stations located in Fengjie, Wanzhou, Qianjiang, Youyang, Liangping, Changshou, Fengdu, Hechuan, Shapingba, Jiangjin, Qijiang and Dazu (Figure 2). This study collected daily concentrations of ambient air pollutants for the period from December 2016 to December 2022 from the China Meteorological Data Sharing Service System, including particulate matter with an aerodynamic diameter ≤2.5 μm or 10 μm (PM_2.5_ and PM_10_, respectively), nitrogen dioxide (NO_2_), sulphur dioxide (SO_2_), carbon monoxide (CO) and ozone (O_3_). Due to the need to protect patient privacy, we were unable to obtain the home addresses of each participant. Therefore, average exposure estimates were calculated using geocoding based on hospital locations, with exposure levels derived from data of the nearest monitoring stations, representing the pollution exposure of pregnant women who gave birth in those hospitals.

### Covariates in the association

Based on a comprehensive review of existing literature, this study systematically collected maternal clinical baseline data to thoroughly evaluate risk factors associated with PTB in twin pregnancies. The analysis was conducted from three primary dimensions: (i) Sociodemographic characteristics: the age of the pregnant women, pre-pregnancy body mass index (PBMI), gravidity, nulliparity, (ii) Maternal baseline characteristics: *in vitro* fertilization (IVF), dichorionic diamniotic (DCDA), scarred uterus; (iii) Independent risk factors for preterm birth: placenta previa, foetal growth restriction (FGR), gestational diabetes (GDM), preeclampsia (PE) [29–31]. Furthermore, the time of conception was categorized into seasons based on the month information as spring, summer, autumn and winter.

## Definition

During the study period, to explore the sensitive windows of exposure to air pollution for PTB in twin pregnancies, we designated the 75th and 85th percentiles of the local air pollutant concentrations as thresholds for assessing high pollution exposure and the 95th percentile as the critical threshold for assessing extreme air pollution exposure. Considering that different regions have distinct environments, which can affect the local population in various ways, this study aimed to establish an index that could be most universally applicable. Based on the definitions of extreme temperature heatwaves and cold waves, the study defined extreme pollution events. Therefore, the extreme pollution threshold was defined in the form of percentiles [29]. To comprehensively reflect scenarios of extreme air pollution exposure, the study further expanded the threshold range to include the 90th percentile, creating four indices of extreme air pollution exposure: 90th-days, 90th-2D, 95th-days and 95th-2D. The 90th-days and 95th-days indices represent the total number of days within a specific exposure window where air pollutant concentrations were at or above the 90th and 95th percentiles, respectively. The 95th-2D and 90th-2D indices represent the frequency of consecutive two-day periods where air pollutant concentrations were at or above the 90th and 95th percentiles, respectively [29]. To further assess the cumulative effects of extreme air pollution exposure on PTB in twin pregnancies at different lag times, we categorized the lag periods into short-term (0–1 lag week and 0–1 lag month) and long-term (0–3 lag months, 0–6 lag months and 0–9 lag months) categories.

### Preterm birth outcomes

Gestational age (GA) was calculated based on the last menstrual period, adjusted by the crown-rump length in the first trimester or the larger foetus’s head circumference after 14 weeks of pregnancy determined by ultrasound and for pregnancies conceived through assisted reproductive technologies, the timing of IVF.

PTB is commonly defined as childbirth occurring before 37 completed weeks of gestation, and this definition is further subdivided into different subtypes. Based on the specific timing of delivery, PTB is categorized into early preterm birth (early-PTB, 24–34 weeks) and late preterm birth (late-PTB, 35–36 weeks) [32]. Furthermore, depending on the specific clinical circumstances of the preterm birth, it can be further subdivided into medically indicated preterm birth, preterm premature rupture of membranes (PPROM) and spontaneous preterm birth [29,33].

Medically indicated preterm birth refers to preterm delivery necessitated by medical or obstetrical complications such as chronic or gestational hypertension, PE, GDM, placenta previa, or placental abruption. PPROM specifically denotes the situation where the foetal membranes rupture prior to the onset of labour without medical intervention. Preterm births that do not fall into the aforementioned categories are classified as spontaneous preterm births.

### Statistical analysis

Data processing and analysis were performed using SPSS software (version 26.0) and R language (version 4.0.2). The study subjects were divided into two groups for comparison based on whether preterm birth occurred. To describe the sociodemographic characteristics of the study subjects, normally distributed continuous variables were presented in the form of mean ± standard deviation (SD), and non-normally distributed data were expressed as the median and interquartile range (IQR).

In the univariate analysis phase, we compared continuous and categorical variables between the preterm birth group and the non-preterm birth group. Categorical variables were described using frequencies and their respective proportions. The Mann-Whitney U test was used to assess differences in continuous variables, while the chi-square test or Fisher’s exact test was used to evaluate differences between groups for categorical variables.

Subsequently, to investigate the relationship between air pollutants (PM_2.5_, PM_10_, SO_2_, NO_2_, CO, O_3_) and the risk of PTB in twin pregnancies, we applied a DLNM after adjusting for the effects of the day of the week and the birth season. We then created three-dimensional lag-dose-response plots to visually display the overall relationship between air pollutant concentrations and PTB risk. Furthermore, the study used DLNMs to calculate the cumulative effects of air pollutant concentrations relative to their 95th percentile exposure during different gestational weeks.

Then, potential confounding factors such as maternal age, PBMI and scarred uterus were adjusted using a multivariate Logistic regression model. This was to assess the cumulative impact of the duration and frequency of exposure to different extreme pollution levels at different times before delivery on the risk of preterm birth in twin pregnancies. Due to the distinct pathophysiological mechanisms associated with different preterm birth subtypes, the study further employed a logistic regression model to investigate the cumulative impact of both the duration and frequency of exposure to varying levels of extreme pollution at different time points prior to delivery.

Finally, preterm births were subdivided into various subtypes according to different criteria, DLNMs were established for each subtype and contour plots of the relationship between air pollutant concentrations and PTB subtype risks were created for sensitivity analysis.

### Distributed lag non-linear model

In the initial phase of our study, we employed the DLNM framework in conjunction with quasi-Poisson regression to analyse the potential impact of PM_2.5_, PM_10_, SO_2_, NO_2_, CO and O_3_ on the incidence of PTB. To dynamically assess the relationship between air pollution and PTB, we constructed a bidimensional exposure-lag-response model using cross-basis functions. To ascertain the optimal fit for our model, we integrated both the Quasi-Akaike Information Criterion (QAIC) and the Quasi-Bayesian Information Criterion (QBIC) into our assessment [34–36], thus determining the most suitable selection for degrees of freedom (df). The finalized model incorporated a natural cubic spline function with four degrees of freedom to capture the exposure-response relationship, while a polynomial function with four degrees of freedom was applied to delineate the lag-response relationship.

In an endeavour to thoroughly explore the temporal structure of air pollution effects, taking into account the latency and duration of preterm labour, we set the maximum lag period at 37 weeks. Our model was further refined by including categorical variables representing the day of the week (Dow) and public holidays, as well as a variable for the seasonal attribution of each day (season). Moreover, to precisely evaluate the influence of extreme temperatures on the risk of PTB across different gestational weeks, we defined the 75th and 85th percentile concentrations of each pollutant as high exposure levels, while the 95th percentile was designated as the threshold for extreme air pollution exposure. The lower quartile concentration of each pollutant was selected as the reference concentration for control comparisons. Ultimately, the study utilized the DLNMs to quantitatively assess the relative risks (RRs) and their 95% confidence intervals (CIs) for twin pregnancy PTB associated with pollutant concentrations at the 75th, 85th and 95th percentiles throughout the 1st to 37th weeks of gestation [29].

### Sensitivity analysis

To ensure the robustness of the study results, multiple sensitivity analyses were conducted. First, to verify the appropriateness of using the 95th percentile as the threshold for extreme pollution exposure, we compared susceptibility periods for high pollution exposure at the 75th and 85th percentiles. Second, considering that different subtypes of preterm birth may have different correlations with exposure to air pollutants, we divided preterm births into early preterm birth (early-PTB, gestational age <34 weeks, *n* = 523) and late preterm birth (late-PTB, gestational age ≥34 weeks, *n* = 1543) and further subdivided based on clinical presentations into medically indicated preterm birth (*n* = 1162), preterm birth due to preterm premature rupture of membranes (PPROM, *n* = 329) and spontaneous preterm birth (*n* = 575).

Subsequently, we independently modelled these preterm subtypes using DLNMs and created contour plots to illustrate the relationship between air pollutant concentrations and the risks associated with the different subtypes of preterm birth. This comprehensive approach was used to fully assess the stability of our study results. Additionally, we adjusted the range of degrees of freedom settings for air pollutants in the DLNMs, varying from 3 to 5, to test whether the consistency and statistical significance of the effect of pollutants on the risk of preterm birth in twin pregnancies would differ under different degrees of freedom settings. Through these sensitivity analyses, we were able to more precisely verify the relationship between air pollution and the risk of preterm birth in twin pregnancies and ensure that the study results are scientifically robust and reliable.

## Result

### Analysis of baseline characteristics of participants

In this study, a total of 3623 twin pregnancies were included, of which 2066 were diagnosed with preterm birth, accounting for 57.02%. Compared to those who did not experience preterm birth, women with preterm deliveries had lower proportions of gravidity, nulliparity, IVF and DCDA, while higher proportions were observed for factors such as scarred uterus, placenta previa, FGR, GA, PE and PROM (*p* < .001; Table 1). Beyond the aforementioned factors, no significant differences were found between preterm birth and other variables such as age, PBMI, family history of hypertension and hyperglycaemia, placental implantation, GBS and season of conception.

**Table 1.** Baseline characteristics of the study cohorts.

Characteristics	Total births (*n* = 3623)	Term births (*n* = 1557)	Preterm births (*n* = 2066)	*p* Value
Age, *M* (IQR)	31.00[28.00,33.00]	31.00[29.00,33.00]	31.00[28.00,33.00]	.160
PBMI, *M* (IQR)	21.300[19.56,23.37]	21.260[19.57,23.23]	21.34[19.56,23.44]	.397
Gravidity, *M* (IQR)	2[1,3]	2[1,3]	2[1,3]	.013*
Nulliparity, *n*(%)	2977(82.17)	1321(84.84)	1656(80.16)	<.001*
IVF, *n*(%)	2590(71.50)	1164(74.80)	1426(69.00)	<.001*
DCDA, *n*(%)	2980(82.25)	1365(87.67)	1615(78.17)	<.001*
Family history of hypertension, *n*(%)	494(13.64)	212(13.62)	282(13.65)	.977
Family history of hyperglycaemia, *n*(%)	229(6.32)	108(6.94)	121(5.86)	.186
Scarred uterus, *n*(%)	328(9.05)	95(6.10)	233(11.28)	<.001*
Placenta previa, *n*(%)	121(3.34)	29(1.86)	92(4.45)	<.001*
Placenta accreta, *n*(%)	592(16.34)	245(15.74)	347(16.80)	.393
FGR, *n*(%)	169(4.67)	57(3.66)	112(5.42)	.013*
GDM, *n*(%)	1024(28.26)	405(26.01)	619(29.96)	.009*
PE, *n*(%)	473(13.06)	143(9.18)	330(15.97)	<.001*
GBS, *n*(%)	39(1.08)	13(0.84)	26(1.26)	.221
PROM, *n*(%)	729(20.12)	62(3.98)	667(32.29)	<.001*
Season of conception				.240
Spring, *n*(%)	752(20.76)	322(20.68)	430(20.81)	
Summer, *n*(%)	907(25.04)	365(23.44)	542(26.23)	
Autumn, *n*(%)	910(25.12)	401(25.76)	509(24.64)	
Winter, *n*(%)	1054(29.09)	469(30.12)	585(28.32)	

Abbreviations: PBMI: pre-pregnancy body mass index; IVF: in-vitro fertilization; DCDA: dichorionic diamniotic; FGR: foetal growth restriction; GDM: gestational diabetes mellitus; PE: preeclampsia; GBS: Group B streptococcal infection; PROM: premature rupture of membranes.

*p < .05.

### Characteristics of extreme air pollution exposure

Air pollution data from the participants’ regions were collected and analyzed to identify critical thresholds for extreme air pollution exposure (Table 2). As reference baseline values, the lower quartiles for PM_2.5_, PM_10_, SO_2_, NO_2_, CO and O_3_ were respectively 22.0 μg/m³, 36.0 μg/m³, 8.0 μg/m³, 21.0 μg/m³, 1.07 mg/m³ and 6.0 μg/m³. The 95th percentile was used as the demarcation point for extreme air pollution exposure, with corresponding values of 86.4 μg/m³, 129.0 μg/m³, 25.0 μg/m³, 54.0 μg/m³, 1.78 mg/m³ and 48.0 μg/m³. Additionally, Spearman correlation analysis depicted in Figure S1 indicates a positive correlation between daily average concentrations of PM_2.5_, PM_10_, SO_2_, NO_2_ and CO throughout the study period, with a particularly notable correlation between PM_2.5_ and PM_10_ (*r* = .95). However, daily average O_3_ concentrations showed a negative correlation with the concentrations of PM_2.5_, PM_10_, SO_2_, NO_2_ and CO.

**Table 2.** Summary characteristics of pollution indicators.

Pollution indicators	Mean	*SD*	Min	5th	10th	15th	25th	50th	75th	85th	90th	95th	Max
PM_2.5_(μg/m^3^)	40.1	23.6	7.0	13.0	18.0	20.0	22.0	35.0	48.0	64.0	71.0	86.4	138.0
PM_10_(μg/m^3^)	63.5	34.0	12.0	21.0	28.0	34.0	36.0	55.0	81.0	95.4	106.0	129.0	189.0
SO_2_(μg/m^3^)	12.4	6.4	4.0	5.0	5.0	6.0	8.0	10.0	17.0	20.0	21.0	25.0	31.0
NO_2_(μg/m^3^)	32.1	12.3	19.0	19.0	19.0	19.0	21.0	33.0	41.0	50.0	54.0	54.0	54.0
CO (mg/m^3^)	1.26	0.32	0.95	1.01	1.01	1.02	1.07	1.27	1.28	1.48	1.48	1.78	2.50
O_3_(μg/m^3^)	15.9	11.2	3.0	5.0	5.0	5.0	6.0	11.0	23.0	24.0	25.0	48.0	48.0

Abbreviations: PM_2.5_: particulate matter with an aerodynamic diameter ≤2.5 μm; PM_10_: particulate matter with an aerodynamic diameter ≤10 μm; SO_2_: sulphur dioxide; NO_2_: nitrogen dioxide; CO: carbon monoxide; O_3_: ozone.

### Identifying the windows of extreme air pollution exposure for PTB in twin pregnancies using DLNM

**Figure 3.**
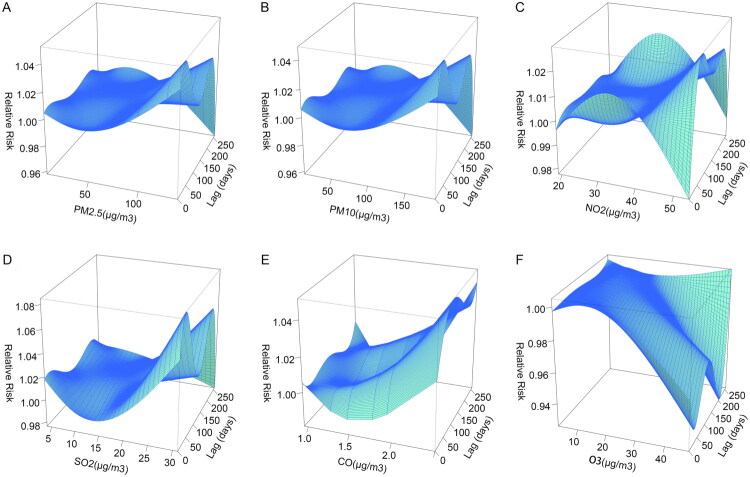
Three-dimensional plot of air pollution versus PTB risk. Distribution lag nonlinear models combined with a quasi-Poisson regression were applied to estimate aRR (95%CI) of PTB with different percentiles of air pollution. The reference level of RR is the 25th percentile of the respective concentrations of air pollutants (PM_2.5_ for 22.0 μg/m^3^, PM_10_ for 36.0 μg/m^3^, NO_2_ for 21.0μg/m^3^, SO_2_ for 8.0 μg/m^3^, CO for 1.07 mg/m^3^, O_3_ for 6.0μg/m^3^). All models were adjusted for the day of week and season. When lag days are 0, it refers to the time of delivery. Abbreviations: PM_2.5_: particulate matter with an aerodynamic diameter ≤2.5μm; PM_10_: particulate matter with an aerodynamic diameter ≤10μm; SO_2_: sulphur dioxide; NO_2_: nitrogen dioxide; CO: carbon monoxide; O_3_: ozone.

Using the DLNM, the lag-dose-response relationships between concentrations of pollutants and the risk of PTB in twin pregnancies were depicted (Figure 3). The results indicated that within specific gestational weeks, higher exposure levels of PM_2.5_, PM_10_, SO_2_, NO_2_ and CO were associated with a bimodal relationship with the risk of PTB in twin pregnancies. This suggests that these pollutants may promote the occurrence of PTB. Consistent with the Spearman analysis results, O_3_ showed a bimodal valley risk trend at higher exposure levels, implying that O_3_ may have a certain inhibitory effect on the occurrence of PTB in twin pregnancies.

**Figure 4.**
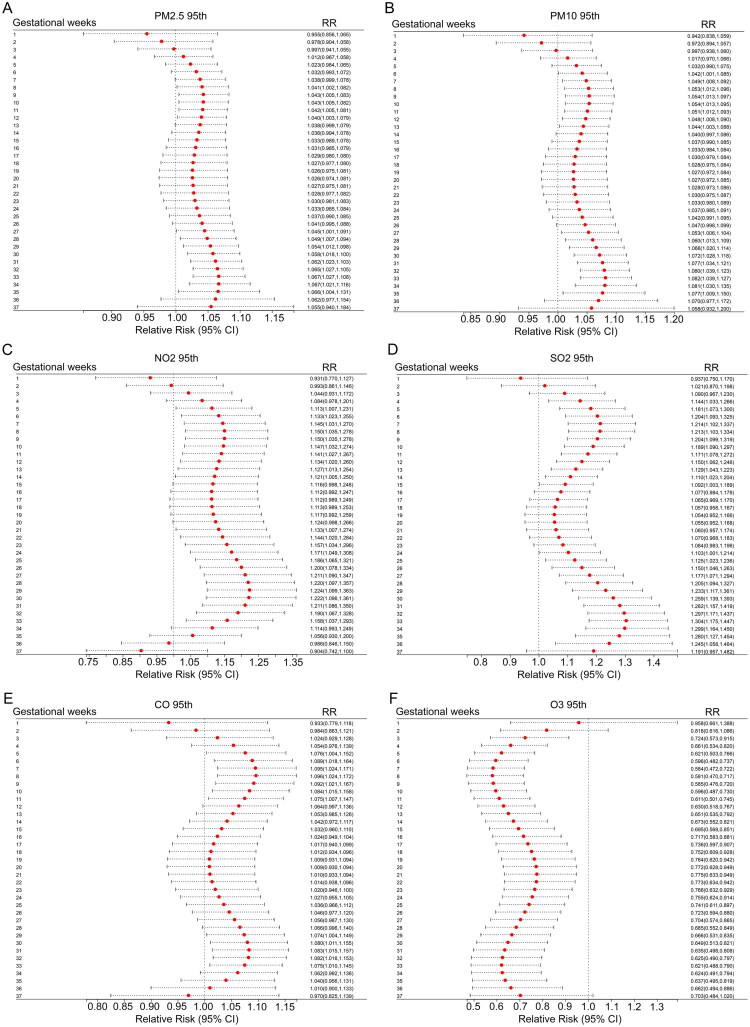
Effect of extreme air pollution exposure on twin pregnancy PTB risk at different gestational weeks. Distribution lag nonlinear models combined with a quasi-Poisson regression were applied to estimate aRR (95%CI) of PTB with different percentiles of air pollution. The reference level of RR is the 25th percentile of the respective concentrations of air pollutants (PM_2.5_ for 22.0 μg/m^3^, PM_10_ for 36.0 μg/m^3^, NO_2_ for 21.0μg/m^3^, SO_2_ for 8.0 μg/m^3^, CO for 1.07 mg/m^3^, O_3_ for 6.0 μg/m^3^). The reference line is X = 1. All models were adjusted for the day of week and season. Abbreviations: PM_2.5_: particulate matter with an aerodynamic diameter ≤2.5μm; PM_10_: particulate matter with an aerodynamic diameter ≤10μm; SO_2_: sulphur dioxide; NO_2_: nitrogen dioxide; CO: carbon monoxide; O_3_: ozone.

To further explore the impact of high pollution concentration exposure on PTB in twin pregnancies, the 95th percentile was used as the threshold for extreme air pollution, and the effect of this extreme exposure on the risk of PTB was assessed at specific gestational week levels (Figure 4). The results showed that the impact patterns of PM_2.5_ and PM_10_ were highly similar; compared to their respective lower quartiles (PM_2.5_: 22.0 μg/m³, PM_10_: 36.0 μg/m³), the 95th percentile exposure of PM_2.5_ was associated with an increased risk of PTB in twin pregnancies between gestational weeks 8 to 12 and 27 to 35, with peak risks at week 9 (aRR and 95% CI: 1.043 [1.005, 1.083]) and week 34 (aRR and 95% CI: 1.067 [1.021, 1.116]), respectively; the 95th percentile exposure of PM_10_ was associated with increased PTB risk between gestational weeks 6 to 13 and 27 to 35, with peak risks at week 9 (aRR and 95% CI: 1.054 [1.013, 1.097]) and week 33 (aRR and 95% CI: 1.082 [1.039, 1.127]).

Compared to PM_2.5_ and PM_10_, NO_2_ and SO_2_ exhibited longer risk exposure windows. For NO_2_, the 95th percentile exposure was positively associated with the risk of PTB in twin pregnancies compared to its lower quartile (54.0 μg/m³) between gestational weeks 5 to 14 and 21 to 33, with peak risks at week 9 (aRR and 95% CI: 1.150 [1.035, 1.278]) and week 28 (aRR and 95% CI: 1.224 [1.099, 1.363]); for SO_2_, the 95th percentile exposure was positively associated with increased PTB risk compared to its lower quartile (8.0 μg/m³) between gestational weeks 4 to 15 and 24 to 36, with peak risks at week 7 (aRR and 95% CI: 1.214 [1.102, 1.337]) and week 31 (aRR and 95% CI: 1.083 [1.015, 1.157]). CO also showed a similar bimodal exposure window. For CO, the 95th percentile exposure was positively associated with an increased risk of PTB in twin pregnancies compared to its lower quartile between gestational weeks 4 to 11 and 29 to 33, with peak risks at week 8 (aRR and 95% CI: 1.096 [1.024, 1.172]) and week 34 (aRR and 95% CI: 1.067 [1.021, 1.116]).

However, the effect of O_3_ on PTB in twin pregnancies was the opposite of other pollutants. Compared to the lower quartile of O_3_, the 95th percentile exposure was associated with a reduced risk of PTB across gestational weeks 3 to 36, with troughs at week 8 (aRR and 95% CI: 0.581 [0.470, 0.717]) and week 33 (aRR and 95% CI: 0.621 [0.488, 0.790]).

In summary, the critical windows for extreme pollution exposure were from gestational weeks 4 to 15 and 21 to 35, particularly concentrated between weeks 8 to 11 and 29 to 33.

### Cumulative effects of extreme air pollution exposure on PTB in twin pregnancies over different lag periods

To dissect the cumulative impact of extreme air pollution on PTB in twin pregnancies, this study innovatively combined the severity of pollutant concentrations with the duration of exposure to define four extreme air pollution indices, drawing inspiration from the definition methods used for heatwaves and cold spells. This approach aimed to more intuitively identify pollution levels associated with high health risks for a more precise assessment of potential risk factors for PTB in twin pregnancies.

Using the 90th percentile as a new extreme pollution threshold, four extreme air pollution indices were constructed (Table 3). The potential impacts of these extreme exposure events on the risk of PTB in twin pregnancies were then thoroughly evaluated from two dimensions: short-term exposure (0–1 week lag and 0–1 month lag) and long-term exposure (0–3 months lag, 0–6 months lag and 0–9 months lag). During the 0–1 week lag period, after adjusting for relevant covariates, exposures other than SO_2_ did not show a positive correlation with the risk of PTB in twin pregnancies (adjusted Odds Ratio [aOR] and 95%CI: SO2: 95th-days: 1.149 [1.032,1.282]).

**Table 3.** Associations between extreme pollution events and preterm birth.

Variables	0–1 lag week		0–1 lag month		0–3 lag months		0–6 lag months		0–9 lag months	
	aOR(95%CI)	*p* Value	aOR(95%CI)	*p* Value	aOR(95%CI)	*p* Value	aOR(95%CI)	*p* Value	aOR(95%CI)	*p* Value
PM_2.5_										
90th-days	1.001(0.947,1.057)	.984	1.019(1.000,1.038)	.047*	1.008(1.001,1.016)	.025*	1.006(1.001,1.011)	.022*	1.006(1.001,1.011)	.014*
90th-2D	0.990(0.923,1.064)	.791	1.031(1.004,1.058)	.023*	1.012(1.002,1.023)	.019*	1.009(1.001,1.016)	.021*	1.008(1.001,1.015)	.017*
95th-days	1.000(0.920,1.088)	.995	1.041(1.010,1.073)	.010*	1.018(1.006,1.031)	.004*	1.014(1.005,1.023)	.002*	1.013(1.005,1.022)	.001*
95th-2D	1.012(0.905,1.134)	0.841	1.059(1.014,1.107)	.011*	1.031(1.012,1.050)	.002*	1.025(1.011,1.039)	<.001*	1.001(0.947,1.057)	.984
PM_10_										
90th-days	1.002(0.954,1.053)	.941	1.018(1.002,1.034)	.031*	1.008(1.001,1.014)	.016*	1.006(1.002,1.010)	.007*	1.006(1.002,1.010)	.004*
90th-2D	0.987(0.926,1.053)	.700	1.023(1.000,1.047)	.047*	1.011(1.002,1.020)	.019*	1.008(1.002,1.015)	.012*	1.009(1.003,1.015)	.006*
95th-days	1.018(0.938,1.106)	.668	1.043(1.013,1.075)	.006*	1.019(1.006,1.031)	.003*	1.014(1.005,1.023)	.002*	1.013(1.005,1.022)	.001*
95th-2D	1.039(0.932,1.162)	.491	1.068(1.023,1.116)	.003*	1.032(1.014,1.052)	.001*	1.026(1.012,1.040)	<.001*	1.002(0.954,1.053)	.941
SO_2_										
90th-days	1.056(0.982,1.135)	.141	1.035(1.010,1.061)	.006*	1.016(1.006,1.026)	.001*	1.012(1.005,1.019)	<.001*	1.012(1.005,1.018)	<.001*
90th-2D	1.013(0.859,1.198)	.876	1.023(0.967,1.083)	.430	1.033(1.008,1.059)	.009*	1.028(1.011,1.045)	.001*	1.027(1.012,1.042)	<.001*
95th-days	1.149(1.032,1.282)	.012*	1.079(1.034,1.126)	<.001*	1.034(1.015,1.053)	<.001*	1.024(1.010,1.038)	.001*	1.024(1.011,1.037)	<.001*
95th-2D	1.295(0.965,1.786)	.097	1.125(0.992,1.280)	.070	1.076(1.019,1.137)	.009*	1.048(1.010,1.088)	.014*	1.056(0.982,1.135)	.141
CO										
90th-days	0.966(0.908,1.028)	.282	0.996(0.970,1.023)	.786	1.001(0.989,1.014)	.846	1.004(0.994,1.013)	.447	1.001(0.993,1.008)	.892
90th-2D	0.959(0.842,1.094)	.533	1.008(0.950,1.071)	.784	1.012(0.983,1.041)	.438	1.024(1.004,1.044)	.016*	1.016(1.000,1.033)	.045*
95th-days	1.037(0.954,1.127)	.393	1.024(0.993,1.056)	.135	1.012(0.999,1.025)	.074	1.010(1.001,1.019)	.023*	1.009(1.002,1.017)	.015*
95th-2D	0.985(0.782,1.243)	.896	1.013(0.918,1.119)	.794	1.004(0.963,1.046)	.865	1.005(0.980,1.030)	.713	0.966(0.908,1.028)	.282
O_3_										
90th-days	1.069(0.987,1.158)	.103	1.048(1.010,1.086)	.012*	1.018(1.000,1.036)	.047*	1.014(1.003,1.025)	.015*	1.012(1.003,1.021)	.007*
90th-2D	1.023(0.824,1.273)	.839	1.029(0.925,1.146)	.597	1.002(0.945,1.062)	.950	1.009(0.974,1.045)	.629	1.006(0.978,1.035)	.678
95th-days	1.104(0.977,1.249)	.112	1.015(0.960,1.074)	.599	1.009(0.977,1.042)	.592	1.017(0.996,1.038)	.107	1.015(0.998,1.031)	.077
95th-2D	0.838(0.428,1.649)	.605	0.968(0.681,1.382)	.857	0.895(0.733,1.093)	.275	1.015(0.910,1.133)	.794	1.069(0.987,1.158)	.103
NO_2_										
90th-days	0.923(0.853,0.999)	.048*	0.958(0.924,0.993)	.019*	0.966(0.946,0.987)	.002*	0.973(0.957,0.990)	.001*	0.967(0.953,0.981)	<.001*
90th-2D	0.865(0.676,1.108)	.249	0.876(0.776,0.989)	.032*	0.934(0.867,1.006)	.071	1.012(0.957,1.071)	.671	0.975(0.926,1.026)	.329
95th-days	0.923(0.853,0.999)	.048*	0.958(0.924,0.993)	.019*	0.966(0.946,0.987)	.002*	0.973(0.957,0.990)	.001*	0.967(0.953,0.981)	<.001*
95th-2D	0.865(0.676,1.108)	.249	0.876(0.776,0.989)	.032*	0.934(0.867,1.006)	.071	1.012(0.957,1.071)	.671	0.923(0.853,0.999)	.048*

*Notes:* Associations between extreme pollution events and preterm birth. Multivariate logistic regression models were applied to estimate aOR (95%CI) of PTB. All models were adjusted for age, PBMI, Gravidity, Nulliparity, IVF, DCDA, scarred uterus, placenta previa, FGR, GDM, PE. 90th-days, 90th-2D, 95th-days and 95th-2D represent the frequency of extreme pollution exposure indices. The 90th-days and 95th-days indices represent the total number of days within a specific exposure window where air pollutant concentrations reach or exceed the 90th and 95th percentiles, respectively, while the 90th-2D and 95th-2D indices indicate the frequency of concentrations reaching or exceeding these percentiles on two consecutive days. When lag days are 0, it refers to the time of delivery.

Abbreviations: PM_2.5_: particulate matter with an aerodynamic diameter ≤2.5 μm; PM_10_: particulate matter with an aerodynamic diameter ≤10 μm; SO_2_: sulphur dioxide; NO_2_: nitrogen dioxide; CO: carbon monoxide; O_3_: ozone; PBMI: Pre-pregnancy Body Mass Index.

*p < .05.

The analysis was further expanded to include the cumulative effects of extreme air pollution exposure over a 0–1 month lag period. The results indicated a consistent positive correlation between cumulative exposure to PM_2.5_, PM_10_ and SO_2_ and an increased risk of PTB (aOR and 95%CI: PM_2.5_: 90th-days: 1.019 [1.000,1.038], 90th-2D: 1.012 [1.002,1.023], 95th-days: 1.041 [1.010,1.073], 95th-2D: 1.059 [1.014,1.107]; PM_10_: 90th-days: 1.018 [1.002,1.034], 90th-2D: 1.023 [1.000,1.047], 95th-days: 1.043 [1.013,1.075], 95th-2D: 1.068 [1.023,1.116]; SO_2_: 90th-days: 1.035 [1.010,1.061], 95th-days: 1.079 [1.034,1.126]). There was no significant association between cumulative exposure to CO and an increased risk of PTB.

Finally, the correlation between long-term extreme pollution exposure and PTB risk was explored, analysing the cumulative effects over 0–3 months lag, 0–6 months lag and 0–9 months lag periods. The results showed a consistent positive correlation between PM_2.5_, PM_10_ and SO_2_ and an increased risk of PTB across different exposure windows (0–9 months lag: aOR and 95%CI: PM_2.5_: 90th-days: 1.006 [1.001, 1.011], 90th-2D: 1.008 [1.001, 1.015], 95th-days: 1.013 [1.005, 1.022]; PM_10_: 90th-days: 1.006 [1.002, 1.010], 90th-2D: 1.009 [1.003, 1.015], 95th-days: 1.013 [1.005, 1.022]; SO_2_: 90th-days: 1.012 [1.005, 1.018], 90th-2D: 1.027 [1.012, 1.042], 95th-days: 1.024 [1.011, 1.037]). Additionally, CO began to show a positive correlation with an increased risk of PTB during the 0–6 months lag period, which persisted into the 0–9 months lag period (0–9 months lag: aOR and 95%CI: 90th-2D: 1.016 [1.000, 1.033], 95th-days: 1.009 [1.002, 1.017]).

It is noteworthy that the performance of O_3_ and NO_2_ in multivariate logistic regression analyses was in stark contrast to the DLNM results. O_3_ showed a strong correlation with PTB risk across different lag periods, but this correlation was only evident at the 90th percentile threshold, which may reflect the instability of the relationship between O_3_ and PTB in twin pregnancies. Surprisingly, NO_2_ exhibited a significant negative correlation with increased PTB risk across all lag exposure windows. The study further explored the association between extreme pollution and the risk of preterm birth in clinical subtypes of twin pregnancies. The specific results can be found in Tables S8–S10.

### Sensitivity analysis

To ensure the stability and reliability of the study results, a multi-faceted sensitivity analysis strategy was adopted. Firstly, by conducting DLNM window analyses for the 75th and 85th percentile exposure levels of air pollutants, all significant correlation results were found to be consistent with the results of the 95th percentile extreme air pollution threshold set in the main analysis (Supplementary Tables S1 to S6). This indicates that the main findings are robust across different exposure levels.

Based on the timing of delivery and clinical phenotypes, preterm births were finely classified, and independent DLNMs were constructed for each subtype. Contour plots of the relationship between the risk of different types of preterm birth and exposure to air pollutants showed that the correlations of risk for different preterm subtypes were generally consistent with the overall risk of preterm birth, further extending the applicability and universality of the study conclusions (Figures S2 to S6). Lastly, to more precisely capture the relationship between air pollutant concentrations and the risk of preterm birth, the range of degrees of freedom settings for air pollutants in the DLNM was adjusted, varying from 3 to 5 degrees of freedom. By comprehensively considering the QAIC and the QBIC, the model was found to fit best with 4 degrees of freedom (Supplementary Table S7). This adjustment ensured the optimization of the model, making the analysis results more precise and credible. Through these sensitivity analyses, not only was the robustness of the study results verified, but the credibility of the conclusions was also reinforced from different perspectives.

## Discussion

Our study indicates that twin pregnancies exhibit a heightened risk of PTB, with a PTB rate of 57.0%, aligning closely with previous findings [37]. Extant literature suggests that air pollutants such as particulate matter, nitrogen oxides and ozone may significantly elevate the rate of PTB through mechanisms like epigenetic modifications, oxidative stress responses and placental dysfunction [38–40]. Given the unique physiological state of twin gestations, which predisposes to adverse outcomes compared to singleton pregnancies [41,42], our study aimed to investigate whether twin pregnancies are more susceptible to the risks of PTB associated with extreme air pollution exposure.

Previous research has primarily focused on the effects of average concentrations of air pollutants during different gestational weeks on PTB risk in singleton pregnancies [31,43,44]. For instance, a meta-analysis assessing the impact of average concentrations of air pollutants across the three trimesters found varying sensitivity windows for PTB risk associated with different pollutants [45]. However, studies have indicated that key exposure windows estimated using DLM may better avoid bias compared to those based on average exposures throughout pregnancy [46]. Qiong Wang et al. identified exposure windows spanning the mid to late pregnancy (18–31 weeks) for PM_2.5_, PM_10_, NO_2_ and O_3_ in a study using DLM in Guangzhou [30], which bears similarity to the bimodal trends (8–11 weeks and 29–33 weeks) for PM_2.5_, PM_10_, SO_2_, NO_2_, CO observed in our study for twin PTB. However, our identified exposure windows in early gestation are earlier, and the differences may be explained by physiological mechanisms and modelling algorithms. Twin pregnancies, relative to singletons, possess distinct physiological mechanisms. A study analysing changes in the placental transcriptome profile in early gestation for IVF-ET pregnancies identified 3405 significantly dysregulated differentially regulated genes and over 50 biological processes and pathways [47]. In our twin cohort, with an IVF rate of 71.5%, this could be one of the reasons for the earlier exposure window in gestation. In late gestation, our identified windows are later. E.J.H. Mulder noted that twin foetuses have higher respiratory activity compared to singletons, especially during 30–40 weeks of gestation [48], which might make twin pregnancies more sensitive to air pollutants at later gestational windows, ultimately leading to PTB. In terms of modelling algorithms, traditional DLMs are based on the assumption of a linear effect between exposure and response [49], but an increasing number of studies reveal the relationship between environmental factors and outcomes as complex non-linear effects using restricted cubic splines (RCS) [50–52]. In this study, to establish DLNM and accurately depict the exposure-response relationship, we employed polynomial functions complemented by natural cubic splines to minimize bias. By contrast, Qiong Wang et al. used only natural cubic splines to construct their DLM in the Guangzhou study. This methodological difference may be a key reason for the divergent results in the analysis of air pollution trends between the two studies [30].

Over the past decade, systematic reviews on the association between air pollution and PTB have focused on exposures to average concentrations of PM_2.5_, PM_10_, NO_2_, O_3_, SO_2_ and CO during different pregnancy periods [45,53,54]. Our study translated these average concentration exposures into the frequency of exceeding extreme pollution thresholds combined with the impact of consecutive exposure days. This definition not only facilitates quantification and aids comprehension among clinicians and pregnant women but also calculates the cumulative effects of extreme air pollution exposure over different lag periods with higher spatiotemporal resolution, thereby reducing bias. Our multivariate logistic regression model revealed a positive correlation between the cumulative effects of extreme pollution (including PM_2.5_, PM_10_, SO_2_, CO and O_3_) and an increased risk of PTB in twins, particularly under long-term lags of 0–3 months, 0–6 months and 0–9 months, demonstrating the strongest correlation. Zhe Sun et al. also found that various air pollutants contribute differently to PTB risk, with PM_2.5_, PM_10_ and SO_2_ having the most significant impact [55], consistent with the significant correlations between PM_2.5_, PM_10_, SO_2_ and PTB risk found in our study. The specific relationship between short-term and long-term exposure to high concentrations of extreme CO and PTB has been less explored. A study involving over 500,000 pregnant women in Chongqing found the risk effects of short-term and long-term exposure to high concentrations of CO on PTB [56], which could explain the risk of extreme CO exposure observed in our study to some extent.

Notably, O_3_ exhibits opposing effect directions in logistic regression versus DLNM. This discrepancy likely stems from DLNM’s refined temporal dimensionality modelling—even in single-pollutant frameworks, long-term lag effects remain vulnerable to interference from strongly negatively correlated copollutants (evidenced by complex photochemical interactions and Spearman correlation analyses). We posit that O_3_’s robust negative correlations with other pollutants may mask its true risk effects, resulting in a spurious protective association in DLNM. This contrasts with findings from a Beijing DLNM study (O_3_ peak: 281.02 μg/m³) reporting a bimodal dose-response curve. Our observed lower O_3_ peak concentration (48.0 μg/m³) in Chongqing suggests that ambient O_3_ at this level is more susceptible to copollutant interference and may not reach the threshold required to trigger preterm birth [57]. Crucially, logistic regression captured O_3_’s true risk effect by focusing on extreme exposure percentiles (e.g. 95th) through the ‘extreme pollution event’ framework. While existing evidence confirms O_3_ induces placental dysfunction *via* oxidative stress [40], studies on its extreme-concentration effects remain limited. Supporting our findings, research on O_3_-telomere interplay demonstrates that moderate concentrations prolong telomeres (reducing preterm birth risk), whereas this protective effect vanishes at extreme exposures [58].

The purported protective association of NO_2_ presents greater mechanistic ambiguity. Although some studies report similar phenomena [59–61], NO_2_ is an established risk factor for preterm birth [45,62], implying its protective correlation may reflect methodological bias or residual confounding. Importantly, large singleton cohort studies by Johnson et al. (*n* > 250,000; peak: 35.5 ppb) and Stieb et al. (*n* ≈ 346,000; peak: 27.8 ppb) [60,61] also reported protective effects, whereas Ji et al.’s Shanghai study (*n* ≈ 25,000; peak: 211.6 μg/m³) demonstrated clear risk [63]. This concentration-dependent divergence suggests a potential U-shaped relationship—linear assumptions in logistic regression may misinterpret the initial descending limb of the U-curve as protective. Although Bagate et al. proposed that NO_2_ might counteract O_3_-induced oxidative stress *via* anti-inflammatory pathways [64], its modulation of placental inflammatory cascades requires experimental validation. In contrast, DLNM’s cross-basis functions reveal genuine non-linear associations between NO_2_ and twin preterm birth at high spatiotemporal resolution.

These findings suggest that healthcare providers should closely monitor the dynamics of air pollutants, especially during critical windows of pregnancy and offer timely personalized health guidance to pregnant women to avoid exposure to high concentrations of air pollutants. Specific measures include advising pregnant women to wear medical masks during periods of severe pollution, increase indoor ventilation and adjust their living environment according to the circumstances. Additionally, for prolonged exposure to high concentrations of air pollutants lasting several days, pregnant women should minimize unnecessary outdoor activities and avoid going out during periods of poor air quality to reduce exposure risks and ensure the health of both mother and baby [57,65,66].

## Conclusion

This study systematically assessed the impact of exposure to extreme air pollution on PTB among twin pregnancies, revealing consistent susceptibility windows for PTB in early (8–11 weeks) and late (29–33 weeks) gestation upon exposure to PM_2.5_, PM_10_, NO_2_, SO_2_ and CO. The cumulative effects of extreme air pollution exposure to PM_2.5_, PM_10_, SO_2_, CO and O_3_ were significantly correlated with an increased risk of PTB in twin pregnancies, particularly under long-term lags of 0–3 months, 0–6 months and 0–9 months. Future research should further explore the specific mechanisms of impact of different types of pollutants and their concentrations on twin pregnancies to provide a scientific basis for the formulation of more refined public health policies. At the same time, healthcare providers should be aware of the potential harm of environmental pollution to twin pregnancies and take timely preventive measures.

## Limitations

This study is a single-centre retrospective analysis, with all baseline information obtained through the healthcare centre’s electronic medical record system and reviewed by professional physicians, providing a theoretical foundation and methodological reference for future multi-centre research. In the future, the study scope can be expanded to include data from multiple regions or centres to enhance the generalizability of the results. Although there are currently several large-scale national studies focusing on the environmental sensitive period in pregnant women, accurately assessing the impact of geographic differences on air pollution and its effects on pregnant women remains a challenge in this field. Some key covariate data were missing from the records, including the mother’s occupation, activity patterns, time spent indoors and outdoors during pregnancy and indoor air quality. These omissions may lead to inaccuracies in the estimation of air pollution exposure. Specifically, factors such as occupation and activity patterns may directly influence the exposure levels of pregnant women. For example, certain occupations may lead to higher exposure to air pollution (e.g. outdoor workers), while activity patterns affect the proportion of time pregnant women spend outdoors versus indoors. The absence of data on indoor air quality may also result in inaccurate estimation of indoor air pollution exposure, thereby affecting the precision of the overall exposure assessment [67]. Another scenario is that pregnant women may spontaneously take adaptive measures and actions to cope with high levels of outdoor air pollution exposure, such as relocating. It has been shown that residential mobility is also associated with misclassification of pregnant women’s environmental exposures [68]. Secondly, due to the lack of specific residential information for pregnant women, it was not possible to accurately match the actual pollution exposure for each woman using the inverse distance weighting (IDW) method. This may have led to partial misclassification of air pollution exposure among pregnant women. Nevertheless, previous studies have shown that there is no significant difference between environmental data estimated using the IDW method and data from nearby meteorological stations [69], suggesting that the actual exposure error may be limited. However, the potential bias of this method, including the clustering effect, still needs to be interpreted with caution, especially when there are discrepancies between exposure estimation and actual residential locations. Future research could consider incorporating clustering around hospitals to reduce exposure misclassification. Moreover, previous studies have also shown that residential mobility is closely related to the misclassification of environmental exposure among pregnant women [68]. Changes in residence may lead to discrepancies between the estimated and actual air pollution exposure among pregnant women, further affecting the accuracy of the results. Therefore, when using hospital geocoding to estimate air pollution exposure, this potential bias must be taken into account. Future studies should further explore the combination of IDW and other methods such as random forest interpolation and develop more accurate environmental exposure models based on consideration of residential mobility and other influencing factors. In this way, we can more accurately assess the health risks of pregnant women under different environmental exposures, thereby providing a more reliable basis for the prediction and intervention of adverse pregnancy outcomes such as preterm birth. In addition, the definition of the extreme pollution threshold in this study was not based on cut-offs calculated using methods such as RCS or GAM (Generalized Additive Models), but rather adapted from previous studies on extreme temperature events and adverse pregnancy outcomes [29]. The validity of this pollution definition remains open to discussion. Future research should more carefully address this issue and adopt a more robust approach to defining extreme pollution thresholds. In studies of risk exposure windows in susceptible populations, Xavier Basagaña and Jose Barrera-Gómez have pointed out that multicollinearity can lead to overestimation or underestimation of the results of DLNM, which is not only reflected among air pollutants but also in terms of lag time [70]. To reduce the bias mentioned by them, we only adopted single-pollutant models for modelling and calculated cumulative effects in DLNM in this study. Although previous studies have indicated that including multiple pollutants in the GAM can reduce the bias of single pollutants, they overlooked the multicollinearity among variables [71–73]. Future studies should further attempt to combine BKMR with DLNM to more precisely analyse the sensitivity windows of susceptible populations to air pollutants. Lastly, in this study after grouping by subtype, the sample sizes for PPROM and iatrogenic preterm birth were small, which compromised the reliability of the DLNM model results. Therefore, to better illustrate the relationship between extreme pollution exposure and preterm birth in twins, we used logistic regression models to separately analyse the three subtypes. Given that different subtypes have distinct pathophysiological mechanisms, exploring the impact of air pollution on each subtype is crucial. Future research should expand the sample size to further investigate the underlying mechanisms. Despite these limitations, this study still provides valuable insights into understanding the potential impact of extreme pollution on the risk of preterm birth in twin pregnancies.

## Supplementary Material

Supplemental Material

Supplemental Material

## Data Availability

To protect patient privacy, all personally identifiable information was removed from the cases, and the data collected were anonymized. These anonymized data can be accessed by contacting the corresponding author, provided there is a reasonable request.
