# Supplementary material for: The impact of extreme air pollution on preterm birth in twin pregnancies: identifying susceptible exposure windows
Source: Ann Med. 2025 Jul 20;57(1):2534854. doi: 10.1080/07853890.2025.2534854 (PMC12278472; doi:10.1080/07853890.2025.2534854)
Supplement: Supplemental Material [file IANN_A_2534854_SM8605.docx]

**Figure S1.** Spearman correlation coefficients between daily air pollutant concentrations.

Abbreviations: PM_2.5_, particulate matter with an aerodynamic diameter ≤ 2.5μm; PM_10_, particulate matter with an aerodynamic diameter ≤ 10μm; SO_2_, sulfur dioxide; NO_2_, nitrogen dioxide; CO, carbon monoxide; O_3_, ozone.

**Figure S2.** Contour map of air pollution and early PTB risk,by distribution lag nonlinear model combined with a quasi-poisson regression. All models were adjusted for the day of week and season. When lag days are 0, it refers to the time of delivery. The color scale on the right side of the contour plot represents the adjusted relative risk of PTB from the lower to the upper limit.

Abbreviations: PM_2.5_, particulate matter with an aerodynamic diameter ≤ 2.5μm; PM_10_, particulate matter with an aerodynamic diameter ≤ 10μm; SO_2_, sulfur dioxide; NO_2_, nitrogen dioxide; CO, carbon monoxide; O_3_, ozone.

**Figure S3.** Contour map of air pollution and late PTB risk,by distribution lag nonlinear model combined with a quasi-poisson regression. All models were adjusted for the day of week and season. When lag days are 0, it refers to the time of delivery.. The color scale on the right side of the contour plot represents the adjusted relative risk of PTB from the lower to the upper limit.

Abbreviations: PM_2.5_, particulate matter with an aerodynamic diameter ≤ 2.5μm; PM_10_, particulate matter with an aerodynamic diameter ≤ 10μm; SO_2_, sulfur dioxide; NO_2_, nitrogen dioxide; CO, carbon monoxide; O_3_, ozone.

**Figure S4.** Contour map of air pollution and iatrogenic PTB risk,by distribution lag nonlinear model combined with a quasi-poisson regression. All models were adjusted for the day of week and season. When lag days are 0, it refers to the time of delivery.. The color scale on the right side of the contour plot represents the adjusted relative risk of PTB from the lower to the upper limit.

Abbreviations: PM_2.5_, particulate matter with an aerodynamic diameter ≤ 2.5μm; PM_10_, particulate matter with an aerodynamic diameter ≤ 10μm; SO_2_, sulfur dioxide; NO_2_, nitrogen dioxide; CO, carbon monoxide; O_3_, ozone.

**Figure S5.** Contour map of air pollution and PPROM risk,by distribution lag nonlinear model combined with a quasi-poisson regression. All models were adjusted for the day of week and season. When lag days are 0, it refers to the time of delivery.. The color scale on the right side of the contour plot represents the adjusted relative risk of PTB from the lower to the upper limit.

Abbreviations: PM_2.5_, particulate matter with an aerodynamic diameter ≤ 2.5μm; PM_10_, particulate matter with an aerodynamic diameter ≤ 10μm; SO_2_, sulfur dioxide; NO_2_, nitrogen dioxide; CO, carbon monoxide; O_3_, ozone.

**Figure S6.** Contour map of air pollution and spontaneous PTB risk,by distribution lag nonlinear model combined with a quasi-poisson regression. All models were adjusted for the day of week and season. When lag days are 0, it refers to the time of delivery.. The color scale on the right side of the contour plot represents the adjusted relative risk of PTB from the lower to the upper limit.

Abbreviations: PM_2.5_, particulate matter with an aerodynamic diameter ≤ 2.5μm; PM_10_, particulate matter with an aerodynamic diameter ≤ 10μm; SO_2_, sulfur dioxide; NO_2_, nitrogen dioxide; CO, carbon monoxide; O_3_, ozone.
