# Supplementary material for: The impact of extreme air pollution on preterm birth in twin pregnancies: identifying susceptible exposure windows
Source: Ann Med. 2025 Jul 20;57(1):2534854. doi: 10.1080/07853890.2025.2534854 (PMC12278472; doi:10.1080/07853890.2025.2534854)
Supplement: Supplemental Material [file IANN_A_2534854_SM9594.zip › Supplemental/Table S1.docx]

**Table S1.** PM_2.5_ exposure and the risk of preterm births at different Gestational week

| Gestational week | 75th | 85th | 95th |
| --- | --- | --- | --- |
| 1 | 1.020(0.936,1.111) | 1.004(0.910,1.107) | 0.955(0.856,1.065) |
| 2 | 1.007(0.945,1.073) | 0.999(0.931,1.073) | 0.978(0.904,1.058) |
| 3 | 0.997(0.949,1.048) | 0.997(0.944,1.052) | 0.997(0.941,1.055) |
| 4 | 0.991(0.949,1.034) | 0.995(0.95,1.042) | 1.012(0.967,1.058) |
| 5 | 0.987(0.947,1.028) | 0.995(0.953,1.039) | 1.023(0.984,1.065) |
| 6 | 0.985(0.945,1.026) | 0.995(0.954,1.038) | 1.032(0.993,1.072) |
| 7 | 0.984(0.945,1.025) | 0.997(0.956,1.039) | 1.038(0.999,1.078) |
| 8 | 0.985(0.947,1.024) | 0.998(0.959,1.040) | 1.041(1.002,1.082)* |
| 9 | 0.987(0.951,1.024) | 1.001(0.963,1.040) | 1.043(1.005,1.083)* |
| 10 | 0.990(0.956,1.024) | 1.003(0.967,1.040) | 1.043(1.005,1.082)* |
| 11 | 0.993(0.962,1.024) | 1.006(0.972,1.040) | 1.042(1.005,1.081)* |
| 12 | 0.996(0.968,1.026) | 1.008(0.976,1.041) | 1.040(1.003,1.079)* |
| 13 | 1.000(0.972,1.028) | 1.011(0.979,1.043) | 1.038(0.999,1.079) |
| 14 | 1.003(0.976,1.031) | 1.013(0.981,1.046) | 1.036(0.994,1.078) |
| 15 | 1.006(0.978,1.035) | 1.015(0.982,1.05) | 1.033(0.989,1.078) |
| 16 | 1.009(0.980,1.039) | 1.017(0.981,1.053) | 1.031(0.985,1.079) |
| 17 | 1.011(0.980,1.042) | 1.018(0.981,1.056) | 1.029(0.980,1.080) |
| 18 | 1.012(0.981,1.044) | 1.019(0.981,1.058) | 1.027(0.977,1.080) |
| 19 | 1.013(0.981,1.045) | 1.019(0.981,1.059) | 1.026(0.975,1.081) |
| 20 | 1.012(0.981,1.044) | 1.019(0.981,1.058) | 1.026(0.974,1.081) |
| 21 | 1.012(0.981,1.043) | 1.018(0.981,1.057) | 1.027(0.975,1.081) |
| 22 | 1.01(0.981,1.040) | 1.017(0.981,1.054) | 1.028(0.977,1.082) |
| 23 | 1.007(0.98,1.036) | 1.015(0.981,1.051) | 1.03(0.981,1.083) |
| 24 | 1.004(0.977,1.032) | 1.013(0.981,1.047) | 1.033(0.985,1.084) |
| 25 | 1.000(0.974,1.028) | 1.011(0.979,1.044) | 1.037(0.990,1.085) |
| 26 | 0.996(0.969,1.025) | 1.008(0.977,1.041) | 1.041(0.995,1.088) |
| 27 | 0.992(0.962,1.022) | 1.005(0.973,1.039) | 1.045(1.001,1.091)* |
| 28 | 0.987(0.955,1.020) | 1.002(0.968,1.038) | 1.049(1.007,1.094)* |
| 29 | 0.982(0.948,1.018) | 0.999(0.963,1.037) | 1.054(1.012,1.098)* |
| 30 | 0.978(0.941,1.016) | 0.997(0.958,1.036) | 1.058(1.018,1.100)* |
| 31 | 0.974(0.936,1.013) | 0.994(0.955,1.035) | 1.062(1.023,1.103)* |
| 32 | 0.970(0.932,1.010) | 0.992(0.952,1.033) | 1.065(1.027,1.105)* |
| 33 | 0.968(0.930,1.007) | 0.990(0.951,1.031) | 1.067(1.027,1.108)* |
| 34 | 0.967(0.928,1.007) | 0.989(0.948,1.032) | 1.067(1.021,1.116)* |
| 35 | 0.967(0.924,1.012) | 0.989(0.941,1.040) | 1.066(1.004,1.131)* |
| 36 | 0.969(0.915,1.027) | 0.990(0.927,1.057) | 1.062(0.977,1.154) |
| 37 | 0.974(0.900,1.053) | 0.992(0.906,1.087) | 1.055(0.940,1.184) |

PM_2.5_ exposure and the risk of PTB in specific gestational weeks. Distribution lag nonlinear model combined with a quasi-poisson regression were applied to estimate aRR (95%CI) of PTB with different percentiles (75^th^, 85^th^, and 95^th^) of PM_2.5_ relative to the 25th percentile (22.0 μg/m^3^); All models were adjusted for the day of week and season; **P* < 0.05.
