# Supplementary material for: The impact of extreme air pollution on preterm birth in twin pregnancies: identifying susceptible exposure windows
Source: Ann Med. 2025 Jul 20;57(1):2534854. doi: 10.1080/07853890.2025.2534854 (PMC12278472; doi:10.1080/07853890.2025.2534854)
Supplement: Supplemental Material [file IANN_A_2534854_SM9594.zip › Supplemental/Table S10.docx]

**Table S10. Associations between extreme pollution events and PPROM.**

| **Variables** | **0-1 lag week** | | **0-1 lag month** | | **0-3 lag months** | | **0-6 lag months** | | **0-9 lag months** | |
| --- | --- | --- | --- | --- | --- | --- | --- | --- | --- | --- |
|  | **aOR(95%CI)** | **p-value** | **aOR(95%CI)** | **p-value** | **aOR(95%CI)** | **p-value** | **aOR(95%CI)** | **p-value** | **aOR(95%CI)** | **p-value** |
| PM_2.5_ |  |  |  |  |  |  |  |  |  |  |
| 90^th^-days | 0.993(0.896,1.092) | 0.889 | 1.019(0.986,1.052) | 0.252 | 1.009(0.996,1.022) | 0.156 | 1.004(0.995,1.013) | 0.410 | 1.004(0.996,1.013) | 0.302 |
| 90^th^-2D | 0.977(0.851,1.105) | 0.721 | 1.030(0.983,1.077) | 0.206 | 1.014(0.995,1.032) | 0.142 | 1.006(0.993,1.020) | 0.342 | 1.006(0.993,1.018) | 0.363 |
| 95^th^-days | 1.024(0.877,1.177) | 0.751 | 1.050(0.997,1.104) | 0.060 | 1.022(1.000,1.043) | 0.044* | 1.009(0.993,1.025) | 0.262 | 1.007(0.992,1.022) | 0.349 |
| 95^th^-2D | 1.060(0.864,1.269) | 0.544 | 1.078(1.000,1.158) | 0.047* | 1.033(0.999,1.066) | 0.051 | 1.015(0.990,1.040) | 0.243 | 0.993(0.896,1.092) | 0.889 |
| PM_10_ |  |  |  |  |  |  |  |  |  |  |
| 90^th^-days | 0.986(0.900,1.075) | 0.760 | 1.019(0.99,1.047) | 0.200 | 1.008(0.997,1.019) | 0.154 | 1.004(0.996,1.012) | 0.346 | 1.004(0.997,1.011) | 0.274 |
| 90^th^-2D | 0.969(0.856,1.085) | 0.601 | 1.023(0.982,1.064) | 0.260 | 1.012(0.996,1.028) | 0.144 | 1.005(0.994,1.017) | 0.359 | 1.006(0.995,1.017) | 0.301 |
| 95^th^-days | 1.020(0.875,1.172) | 0.788 | 1.048(0.995,1.102) | 0.070 | 1.023(1.001,1.044) | 0.035* | 1.010(0.994,1.025) | 0.234 | 1.008(0.993,1.023) | 0.289 |
| 95^th^-2D | 1.086(0.896,1.289) | 0.366 | 1.080(1.002,1.158) | 0.038* | 1.034(1.002,1.067) | 0.033* | 1.016(0.992,1.040) | 0.196 | 0.986(0.900,1.075) | 0.760 |
| SO_2_ |  |  |  |  |  |  |  |  |  |  |
| 90^th^-days | 1.037(0.909,1.175) | 0.580 | 1.031(0.987,1.076) | 0.158 | 1.014(0.996,1.032) | 0.113 | 1.006(0.994,1.019) | 0.325 | 1.005(0.993,1.016) | 0.424 |
| 90^th^-2D | 0.825(0.557,1.150) | 0.295 | 0.972(0.867,1.078) | 0.602 | 1.016(0.971,1.061) | 0.468 | 1.009(0.978,1.040) | 0.558 | 1.003(0.975,1.031) | 0.829 |
| 95^th^-days | 1.200(0.992,1.441) | 0.054 | 1.069(0.990,1.151) | 0.083 | 1.026(0.992,1.060) | 0.131 | 1.008(0.983,1.033) | 0.531 | 1.006(0.983,1.030) | 0.595 |
| 95^th^-2D | 1.197(0.660,1.977) | 0.506 | 0.970(0.739,1.236) | 0.815 | 1.008(0.906,1.114) | 0.879 | 0.985(0.915,1.056) | 0.679 | 1.037(0.909,1.175) | 0.580 |
| CO |  |  |  |  |  |  |  |  |  |  |
| 90^th^-days | 0.984(0.879,1.099) | 0.771 | 1.000(0.951,1.050) | 0.984 | 1.010(0.987,1.033) | 0.406 | 1.007(0.991,1.023) | 0.409 | 1.004(0.991,1.018) | 0.524 |
| 90^th^-2D | 0.986(0.775,1.234) | 0.902 | 1.015(0.907,1.131) | 0.790 | 1.011(0.958,1.065) | 0.692 | 1.030(0.996,1.065) | 0.080 | 1.013(0.985,1.042) | 0.353 |
| 95^th^-days | 1.027(0.885,1.184) | 0.723 | 1.027(0.971,1.085) | 0.343 | 1.017(0.994,1.04) | 0.135 | 1.007(0.991,1.022) | 0.405 | 1.008(0.994,1.022) | 0.253 |
| 95^th^-2D | 0.977(0.634,1.445) | 0.913 | 1.045(0.874,1.240) | 0.618 | 1.020(0.946,1.095) | 0.603 | 1.017(0.973,1.062) | 0.445 | 0.984(0.879,1.099) | 0.771 |
| O_3_ |  |  |  |  |  |  |  |  |  |  |
| 90^th^-days | 0.985(0.849,1.136) | 0.836 | 1.048(0.982,1.117) | 0.157 | 1.016(0.984,1.049) | 0.334 | 1.008(0.988,1.029) | 0.414 | 1.009(0.993,1.025) | 0.266 |
| 90^th^-2D | 0.910(0.590,1.348) | 0.653 | 1.143(0.943,1.378) | 0.165 | 1.023(0.919,1.136) | 0.680 | 1.016(0.952,1.083) | 0.625 | 1.018(0.967,1.072) | 0.491 |
| 95^th^-days | 1.140(0.916,1.409) | 0.233 | 1.087(0.985,1.199) | 0.096 | 1.026(0.967,1.088) | 0.388 | 1.018(0.982,1.056) | 0.321 | 1.020(0.991,1.049) | 0.166 |
| 95^th^-2D | 0.770(0.179,2.321) | 0.680 | 1.170(0.630,2.045) | 0.598 | 0.903(0.620,1.286) | 0.583 | 1.099(0.904,1.324) | 0.330 | 0.985(0.849,1.136) | 0.836 |
| NO_2_ |  |  |  |  |  |  |  |  |  |  |
| 90^th^-days | 0.974(0.844,1.119) | 0.710 | 0.974(0.913,1.038) | 0.427 | 0.975(0.938,1.014) | 0.206 | 1.000(0.970,1.032) | 0.980 | 0.988(0.961,1.015) | 0.378 |
| 90^th^-2D | 1.161(0.756,1.722) | 0.475 | 0.947(0.760,1.166) | 0.615 | 0.909(0.792,1.040) | 0.172 | 1.067(0.965,1.179) | 0.205 | 0.968(0.880,1.065) | 0.508 |
| 95^th^-days | 0.974(0.844,1.119) | 0.710 | 0.974(0.913,1.038) | 0.427 | 0.975(0.938,1.014) | 0.206 | 1.000(0.970,1.032) | 0.98 | 0.988(0.961,1.015) | 0.378 |
| 95^th^-2D | 1.161(0.756,1.722) | 0.475 | 0.947(0.76,1.166) | 0.615 | 0.909(0.792,1.040) | 0.172 | 1.067(0.965,1.179) | 0.205 | 0.968(0.880,1.065) | 0.508 |

Associations between extreme pollution events and PPROM. Multivariate logistic regression models were applied to estimate aOR (95%CI) of PTB. All models were adjusted for age, PBMI, Gravidity, Nulliparity, IVF, DCDA, scarred uterus, placenta previa, FGR, GDM, PE. 90th-days, 90th-2D, 95th-days and 95th-2D represent the frequency of extreme pollution exposure indices. The 90th-days and 95th-days indices represent the total number of days within a specific exposure window where air pollutant concentrations reach or exceed the 90th and 95th percentiles, respectively, while the 90th-2D and 95th-2D indices indicate the frequency of concentrations reaching or exceeding these percentiles on two consecutive days. When lag days are 0, it refers to the time of delivery.

Abbreviations:PM_2.5_, particulate matter with an aerodynamic diameter ≤ 2.5μm; PM_10_, particulate matter with an aerodynamic diameter ≤ 10μm; SO_2_, sulfur dioxide; NO_2_, nitrogen dioxide; CO, carbon monoxide; O_3_, ozone; PBMI, Pre-pregnancy Body Mass Index. ****P* < 0.05.**
