# Supplementary material for: The impact of extreme air pollution on preterm birth in twin pregnancies: identifying susceptible exposure windows
Source: Ann Med. 2025 Jul 20;57(1):2534854. doi: 10.1080/07853890.2025.2534854 (PMC12278472; doi:10.1080/07853890.2025.2534854)
Supplement: Supplemental Material [file IANN_A_2534854_SM9594.zip › Supplemental/Table S2.docx]

**Table S2.** PM_10_ exposure and the risk of preterm births at different Gestational week

| Gestational week | 75th | 85th | 95th |
| --- | --- | --- | --- |
| 1 | 1.019(0.931,1.115) | 1.004(0.914,1.102) | 0.942(0.838,1.059) |
| 2 | 1.006(0.942,1.075) | 0.999(0.934,1.070) | 0.972(0.894,1.057) |
| 3 | 0.997(0.946,1.050) | 0.996(0.946,1.050) | 0.997(0.938,1.060) |
| 4 | 0.990(0.946,1.036) | 0.995(0.951,1.040) | 1.017(0.97,1.066) |
| 5 | 0.987(0.944,1.031) | 0.995(0.953,1.038) | 1.032(0.99,1.075) |
| 6 | 0.985(0.943,1.029) | 0.995(0.954,1.038) | 1.042(1.001,1.085)* |
| 7 | 0.985(0.943,1.028) | 0.997(0.956,1.039) | 1.049(1.008,1.092)* |
| 8 | 0.986(0.946,1.028) | 0.998(0.959,1.040) | 1.053(1.012,1.096)* |
| 9 | 0.988(0.950,1.028) | 1.001(0.963,1.040) | 1.054(1.013,1.097)* |
| 10 | 0.991(0.956,1.028) | 1.003(0.968,1.040) | 1.054(1.013,1.095)* |
| 11 | 0.995(0.962,1.029) | 1.006(0.973,1.040) | 1.051(1.012,1.093)* |
| 12 | 0.999(0.968,1.030) | 1.009(0.977,1.041) | 1.048(1.008,1.090)* |
| 13 | 1.002(0.973,1.033) | 1.011(0.981,1.043) | 1.044(1.003,1.088)* |
| 14 | 1.006(0.977,1.036) | 1.014(0.982,1.046) | 1.040(0.997,1.086) |
| 15 | 1.009(0.979,1.040) | 1.016(0.983,1.049) | 1.037(0.990,1.085) |
| 16 | 1.012(0.981,1.044) | 1.017(0.983,1.052) | 1.033(0.984,1.084) |
| 17 | 1.014(0.982,1.047) | 1.019(0.983,1.055) | 1.030(0.979,1.084) |
| 18 | 1.015(0.982,1.049) | 1.019(0.983,1.057) | 1.028(0.975,1.084) |
| 19 | 1.016(0.982,1.050) | 1.019(0.983,1.058) | 1.027(0.972,1.084) |
| 20 | 1.015(0.982,1.049) | 1.019(0.983,1.057) | 1.027(0.972,1.085) |
| 21 | 1.014(0.982,1.047) | 1.018(0.983,1.055) | 1.028(0.973,1.086) |
| 22 | 1.012(0.981,1.044) | 1.017(0.983,1.052) | 1.030(0.975,1.087) |
| 23 | 1.009(0.980,1.039) | 1.015(0.983,1.049) | 1.033(0.980,1.089) |
| 24 | 1.006(0.978,1.035) | 1.013(0.982,1.045) | 1.037(0.985,1.091) |
| 25 | 1.002(0.974,1.030) | 1.010(0.980,1.041) | 1.042(0.991,1.095) |
| 26 | 0.997(0.968,1.027) | 1.007(0.977,1.039) | 1.047(0.998,1.099) |
| 27 | 0.992(0.962,1.024) | 1.004(0.973,1.037) | 1.053(1.006,1.104)* |
| 28 | 0.987(0.954,1.022) | 1.001(0.968,1.035) | 1.060(1.013,1.109)* |
| 29 | 0.982(0.946,1.019) | 0.998(0.962,1.034) | 1.066(1.020,1.114)* |
| 30 | 0.978(0.939,1.017) | 0.994(0.958,1.033) | 1.072(1.028,1.118)* |
| 31 | 0.973(0.934,1.014) | 0.992(0.954,1.031) | 1.077(1.034,1.121)* |
| 32 | 0.970(0.930,1.011) | 0.989(0.951,1.028) | 1.080(1.039,1.123)* |
| 33 | 0.967(0.928,1.008) | 0.987(0.95,1.026) | 1.082(1.039,1.127)* |
| 34 | 0.966(0.927,1.008) | 0.986(0.947,1.027) | 1.081(1.03,1.135)* |
| 35 | 0.967(0.923,1.013) | 0.986(0.941,1.034) | 1.077(1.009,1.15)* |
| 36 | 0.970(0.914,1.029) | 0.987(0.928,1.050) | 1.070(0.977,1.172) |
| 37 | 0.975(0.899,1.058) | 0.990(0.908,1.079) | 1.058(0.932,1.200) |

PM_10_ exposure and the risk of PTB in specific gestational weeks, Distribution lag nonlinear models combined with a quasi-poisson regression were applied to estimate aRR (95%CI) of PTB with different percentiles (75^th^, 85^th^, and 95^th^) of PM_10_ relative to the 25^th^ percentile (36.0 μg/m^3^); All models were adjusted for the day of week and season; **P* < 0.05
