# Supplementary material for: The impact of extreme air pollution on preterm birth in twin pregnancies: identifying susceptible exposure windows
Source: Ann Med. 2025 Jul 20;57(1):2534854. doi: 10.1080/07853890.2025.2534854 (PMC12278472; doi:10.1080/07853890.2025.2534854)
Supplement: Supplemental Material [file IANN_A_2534854_SM9594.zip › Supplemental/Table S3.docx]

**Table S3.** NO_2_ exposure and the risk of preterm births at different Gestational week

| Gestational week | 75^th^ | 85^th^ | 95^th^ |
| --- | --- | --- | --- |
| 1 | 1.092(0.946,1.261) | 0.986(0.853,1.140) | 0.931(0.770,1.127) |
| 2 | 1.077(0.971,1.193) | 1.023(0.919,1.138) | 0.993(0.861,1.146) |
| 3 | 1.064(0.987,1.148) | 1.053(0.969,1.143) | 1.044(0.931,1.172) |
| 4 | 1.055(0.992,1.121) | 1.075(1.002,1.153)* | 1.084(0.978,1.201) |
| 5 | 1.047(0.990,1.107) | 1.091(1.020,1.167)* | 1.113(1.007,1.231)* |
| 6 | 1.041(0.986,1.100) | 1.102(1.030,1.179)* | 1.133(1.023,1.255)* |
| 7 | 1.038(0.982,1.096) | 1.108(1.034,1.187)* | 1.145(1.031,1.270)* |
| 8 | 1.035(0.981,1.092) | 1.110(1.035,1.190)* | 1.150(1.035,1.278)* |
| 9 | 1.034(0.982,1.087) | 1.109(1.034,1.190)* | 1.150(1.035,1.278)* |
| 10 | 1.033(0.985,1.083) | 1.107(1.032,1.188)* | 1.147(1.032,1.274)* |
| 11 | 1.033(0.988,1.080) | 1.103(1.028,1.185)* | 1.141(1.027,1.267)* |
| 12 | 1.034(0.991,1.079) | 1.099(1.023,1.182)* | 1.134(1.020,1.260)* |
| 13 | 1.035(0.993,1.079) | 1.095(1.017,1.180)* | 1.127(1.013,1.254)* |
| 14 | 1.037(0.993,1.082) | 1.092(1.011,1.179)* | 1.121(1.005,1.250)* |
| 15 | 1.038(0.993,1.085) | 1.089(1.006,1.18)* | 1.116(0.998,1.248) |
| 16 | 1.039(0.991,1.090) | 1.088(1.002,1.181)* | 1.112(0.992,1.247) |
| 17 | 1.041(0.99,1.095) | 1.088(0.999,1.184) | 1.112(0.989,1.249) |
| 18 | 1.042(0.989,1.098) | 1.089(0.999,1.188) | 1.113(0.989,1.253) |
| 19 | 1.043(0.988,1.101) | 1.092(1.001,1.192)* | 1.117(0.992,1.259) |
| 20 | 1.044(0.989,1.101) | 1.097(1.005,1.197)* | 1.124(0.998,1.266) |
| 21 | 1.044(0.990,1.101) | 1.103(1.012,1.202)* | 1.133(1.007,1.274)* |
| 22 | 1.044(0.991,1.100) | 1.110(1.020,1.208)* | 1.144(1.020,1.284)* |
| 23 | 1.044(0.993,1.097) | 1.118(1.030,1.213)* | 1.157(1.034,1.296)* |
| 24 | 1.043(0.994,1.095) | 1.127(1.041,1.219)* | 1.171(1.049,1.308)* |
| 25 | 1.042(0.994,1.093) | 1.135(1.052,1.225)* | 1.186(1.065,1.321*) |
| 26 | 1.041(0.993,1.092) | 1.144(1.062,1.231)* | 1.200(1.078,1.334)* |
| 27 | 1.040(0.991,1.091) | 1.151(1.070,1.237)* | 1.211(1.090,1.347)* |
| 28 | 1.039(0.988,1.092) | 1.156(1.076,1.241)* | 1.220(1.097,1.357)* |
| 29 | 1.038(0.985,1.094) | 1.157(1.078,1.243)* | 1.224(1.099,1.363)* |
| 30 | 1.037(0.982,1.094) | 1.156(1.077,1.240)* | 1.222(1.096,1.361)* |
| 31 | 1.036(0.981,1.095) | 1.149(1.071,1.232)* | 1.211(1.086,1.350)* |
| 32 | 1.036(0.981,1.095) | 1.136(1.059,1.219)* | 1.190(1.067,1.328)* |
| 33 | 1.037(0.982,1.095) | 1.116(1.039,1.199)* | 1.158(1.037,1.293)* |
| 34 | 1.039(0.980,1.101) | 1.088(1.009,1.174)* | 1.114(0.993,1.249) |
| 35 | 1.041(0.971,1.117) | 1.052(0.964,1.149) | 1.056(0.930,1.200) |
| 36 | 1.045(0.951,1.148) | 1.007(0.902,1.125) | 0.986(0.846,1.150) |
| 37 | 1.051(0.921,1.199) | 0.953(0.824,1.103) | 0.904(0.742,1.100) |

NO_2_ exposure and the risk of PTB in specific gestational weeks. Distribution lag nonlinear model combined with a quasi-poisson regression were applied to estimate aRR (95%CI) of PTB with different percentiles (75^th^, 85^th^, and 95^th^) of NO_2_ relative to the 25^th^ percentile (21.0μg/m^3^); All models were adjusted for the day of week and season; * *P*<0.05
