# Supplementary material for: The impact of extreme air pollution on preterm birth in twin pregnancies: identifying susceptible exposure windows
Source: Ann Med. 2025 Jul 20;57(1):2534854. doi: 10.1080/07853890.2025.2534854 (PMC12278472; doi:10.1080/07853890.2025.2534854)
Supplement: Supplemental Material [file IANN_A_2534854_SM9594.zip › Supplemental/Table S4.docx]

**Table S4.** SO_2_ exposure and the risk of preterm births at different Gestational week

| Gestational week | 75^th^ | 85^th^ | 95^th^ |
| --- | --- | --- | --- |
| 1 | 0.964(0.891,1.043) | 0.953(0.865,1.051) | 0.937(0.750,1.170) |
| 2 | 0.974(0.919,1.032) | 0.986(0.919,1.057) | 1.021(0.870,1.198) |
| 3 | 0.982(0.939,1.027) | 1.012(0.962,1.064) | 1.090(0.967,1.230) |
| 4 | 0.989(0.953,1.027) | 1.032(0.991,1.074) | 1.144(1.033,1.266)* |
| 5 | 0.995(0.961,1.030) | 1.046(1.010,1.084)* | 1.181(1.073,1.300)* |
| 6 | 0.999(0.966,1.033) | 1.056(1.020,1.093)* | 1.204(1.093,1.325)* |
| 7 | 1.003(0.970,1.036) | 1.061(1.025,1.099)* | 1.214(1.102,1.337)* |
| 8 | 1.005(0.973,1.038) | 1.063(1.026,1.101)* | 1.213(1.103,1.334)* |
| 9 | 1.007(0.976,1.039) | 1.062(1.026,1.099)* | 1.204(1.099,1.319)* |
| 10 | 1.008(0.979,1.039) | 1.059(1.024,1.096)* | 1.189(1.090,1.297)* |
| 11 | 1.009(0.981,1.038) | 1.055(1.021,1.09)* | 1.171(1.078,1.272)* |
| 12 | 1.009(0.982,1.038) | 1.050(1.016,1.085)* | 1.150(1.062,1.246)* |
| 13 | 1.009(0.982,1.037) | 1.044(1.009,1.08)* | 1.129(1.043,1.223)* |
| 14 | 1.009(0.981,1.037) | 1.038(1.002,1.076)* | 1.11(1.023,1.204)* |
| 15 | 1.008(0.980,1.038) | 1.033(0.995,1.073) | 1.092(1.003,1.189)* |
| 16 | 1.008(0.978,1.038) | 1.028(0.988,1.070) | 1.077(0.984,1.178) |
| 17 | 1.007(0.977,1.038) | 1.024(0.982,1.068) | 1.065(0.969,1.170) |
| 18 | 1.006(0.975,1.038) | 1.021(0.978,1.067) | 1.057(0.958,1.167) |
| 19 | 1.005(0.974,1.038) | 1.020(0.975,1.066) | 1.054(0.952,1.166) |
| 20 | 1.004(0.973,1.037) | 1.019(0.975,1.066) | 1.055(0.952,1.168) |
| 21 | 1.004(0.973,1.035) | 1.020(0.976,1.067) | 1.060(0.957,1.174) |
| 22 | 1.003(0.973,1.034) | 1.022(0.979,1.068) | 1.070(0.968,1.183) |
| 23 | 1.002(0.973,1.032) | 1.026(0.983,1.070) | 1.084(0.983,1.196) |
| 24 | 1.001(0.973,1.030) | 1.030(0.989,1.073) | 1.103(1.001,1.214)* |
| 25 | 1.000(0.972,1.029) | 1.035(0.996,1.076) | 1.125(1.023,1.236)* |
| 26 | 0.999(0.971,1.028) | 1.041(1.003,1.081)* | 1.150(1.046,1.263)* |
| 27 | 0.998(0.969,1.027) | 1.047(1.010,1.086)* | 1.177(1.071,1.294)* |
| 28 | 0.996(0.967,1.026) | 1.053(1.017,1.091)* | 1.205(1.094,1.327)* |
| 29 | 0.994(0.964,1.025) | 1.059(1.023,1.096)* | 1.233(1.117,1.361)* |
| 30 | 0.992(0.961,1.024) | 1.064(1.028,1.100)* | 1.259(1.139,1.393)* |
| 31 | 0.989(0.958,1.021) | 1.067(1.032,1.103)* | 1.282(1.157,1.419)* |
| 32 | 0.985(0.954,1.017) | 1.067(1.033,1.103)* | 1.297(1.171,1.437)* |
| 33 | 0.981(0.950,1.013) | 1.065(1.030,1.102)* | 1.304(1.175,1.447)* |
| 34 | 0.976(0.943,1.010) | 1.060(1.020,1.102)* | 1.299(1.164,1.450)* |
| 35 | 0.970(0.930,1.011) | 1.050(0.999,1.104) | 1.280(1.127,1.454)* |
| 36 | 0.962(0.912,1.016) | 1.035(0.966,1.109) | 1.245(1.058,1.464)* |
| 37 | 0.954(0.885,1.027) | 1.015(0.922,1.117) | 1.191(0.957,1.482) |

SO_2_ exposure and the risk of PTB in specific gestational weeks. Distribution lag nonlinear model combined with a quasi-poisson regression were applied to estimate aRR (95%CI) of PTB with different percentiles (75^th^,85^th^, and 95^th^) of SO_2_ relative to the 25^th^ percentile (8.0 μg/m^3^); All models were adjusted for the day of week and season; * *P* < 0.05
