# Supplementary material for: The impact of extreme air pollution on preterm birth in twin pregnancies: identifying susceptible exposure windows
Source: Ann Med. 2025 Jul 20;57(1):2534854. doi: 10.1080/07853890.2025.2534854 (PMC12278472; doi:10.1080/07853890.2025.2534854)
Supplement: Supplemental Material [file IANN_A_2534854_SM9594.zip › Supplemental/Table S5.docx]

**Table S5.** CO exposure and the risk of preterm births at different Gestational week

| Gestational week | 75th | 85th | 95th |
| --- | --- | --- | --- |
| 1 | 0.918(0.837,1.008) | 0.892(0.772,1.031) | 0.933(0.779,1.118) |
| 2 | 0.950(0.884,1.021) | 0.939(0.842,1.048) | 0.984(0.863,1.121) |
| 3 | 0.975(0.920,1.034) | 0.977(0.896,1.066) | 1.024(0.929,1.128) |
| 4 | 0.994(0.944,1.046) | 1.006(0.933,1.084) | 1.054(0.976,1.139)* |
| 5 | 1.007(0.959,1.057) | 1.025(0.957,1.099) | 1.076(1.004,1.152)* |
| 6 | 1.015(0.968,1.064) | 1.038(0.970,1.11) | 1.089(1.018,1.164)* |
| 7 | 1.018(0.972,1.067) | 1.043(0.977,1.114) | 1.095(1.024,1.171)* |
| 8 | 1.018(0.973,1.066) | 1.044(0.978,1.113) | 1.096(1.024,1.172)* |
| 9 | 1.016(0.972,1.061) | 1.039(0.976,1.107) | 1.092(1.021,1.167)* |
| 10 | 1.011(0.969,1.054) | 1.032(0.971,1.097) | 1.084(1.015,1.158)* |
| 11 | 1.004(0.964,1.047) | 1.023(0.963,1.086) | 1.075(1.007,1.147)* |
| 12 | 0.997(0.958,1.039) | 1.012(0.954,1.074) | 1.064(0.997,1.136) |
| 13 | 0.990(0.950,1.031) | 1.001(0.942,1.062) | 1.053(0.985,1.126) |
| 14 | 0.982(0.942,1.024) | 0.990(0.93,1.052) | 1.042(0.972,1.117) |
| 15 | 0.975(0.935,1.018) | 0.979(0.919,1.044) | 1.032(0.960,1.110) |
| 16 | 0.969(0.927,1.013) | 0.97(0.908,1.036) | 1.024(0.949,1.104) |
| 17 | 0.964(0.921,1.009) | 0.963(0.899,1.031) | 1.017(0.94,1.099) |
| 18 | 0.960(0.916,1.006) | 0.957(0.893,1.026) | 1.012(0.934,1.096) |
| 19 | 0.957(0.913,1.003) | 0.953(0.888,1.023) | 1.009(0.931,1.094) |
| 20 | 0.956(0.912,1.002) | 0.952(0.887,1.021) | 1.009(0.93,1.094) |
| 21 | 0.956(0.913,1.002) | 0.952(0.888,1.021) | 1.01(0.933,1.094) |
| 22 | 0.958(0.915,1.002) | 0.955(0.892,1.022) | 1.014(0.938,1.096) |
| 23 | 0.960(0.918,1.004) | 0.959(0.897,1.025) | 1.020(0.946,1.100) |
| 24 | 0.964(0.923,1.008) | 0.966(0.905,1.031) | 1.027(0.955,1.105) |
| 25 | 0.969(0.928,1.013) | 0.973(0.913,1.038) | 1.036(0.966,1.112) |
| 26 | 0.975(0.934,1.019) | 0.982(0.922,1.047) | 1.046(0.977,1.120) |
| 27 | 0.982(0.939,1.026) | 0.992(0.930,1.057) | 1.056(0.987,1.130) |
| 28 | 0.988(0.944,1.034) | 1.001(0.938,1.069) | 1.066(0.996,1.140) |
| 29 | 0.994(0.949,1.042) | 1.010(0.945,1.080) | 1.074(1.004,1.149)* |
| 30 | 0.999(0.953,1.048) | 1.018(0.952,1.089) | 1.080(1.011,1.155)* |
| 31 | 1.003(0.956,1.053) | 1.023(0.956,1.094) | 1.083(1.015,1.157)* |
| 32 | 1.005(0.958,1.054) | 1.025(0.959,1.096) | 1.082(1.016,1.153)* |
| 33 | 1.004(0.958,1.053) | 1.022(0.958,1.092) | 1.075(1.01,1.145)* |
| 34 | 1.000(0.954,1.048) | 1.014(0.949,1.084) | 1.062(0.992,1.136) |
| 35 | 0.992(0.943,1.043) | 1.000(0.929,1.077) | 1.040(0.956,1.131) |
| 36 | 0.978(0.921,1.040) | 0.978(0.892,1.072) | 1.010(0.900,1.133) |
| 37 | 0.960(0.886,1.039) | 0.947(0.837,1.072) | 0.970(0.825,1.139) |

CO exposure and the risk of PTB in specific gestational weeks. Distribution lag nonlinear model combined with a quasi-poisson regression were applied to estimate aRR(95%CI) of PTB with different percentiles (75^th^, 85^th^, and 95^th^) of CO relative to the 25^th^ percentile (1.07 mg/m3); All models were adjusted for the day of week and season; * *P*<0.05
