# Supplementary material for: The impact of extreme air pollution on preterm birth in twin pregnancies: identifying susceptible exposure windows
Source: Ann Med. 2025 Jul 20;57(1):2534854. doi: 10.1080/07853890.2025.2534854 (PMC12278472; doi:10.1080/07853890.2025.2534854)
Supplement: Supplemental Material [file IANN_A_2534854_SM9594.zip › Supplemental/Table S6.docx]

**Table S6.** O_3_ exposure and the risk of preterm births at different Gestational week

| Gestational week | 75^th^ | 85^th^ | 95^th^ |
| --- | --- | --- | --- |
| 1 | 0.964(0.826,1.126) | 0.963(0.823,1.127) | 0.958(0.661,1.388) |
| 2 | 0.955(0.855,1.067) | 0.951(0.850,1.064) | 0.818(0.616,1.086) |
| 3 | 0.949(0.875,1.029) | 0.942(0.867,1.023) | 0.724(0.573,0.915)* |
| 4 | 0.945(0.886,1.008) | 0.936(0.876,1.000) | 0.661(0.534,0.820)* |
| 5 | 0.943(0.889,1.001) | 0.933(0.878,0.992)* | 0.621(0.503,0.766)* |
| 6 | 0.943(0.889,1.001) | 0.932(0.878,0.990)* | 0.596(0.482,0.737)* |
| 7 | 0.945(0.890,1.003) | 0.933(0.878,0.992)* | 0.584(0.472,0.722)* |
| 8 | 0.947(0.893,1.005) | 0.935(0.88,0.994)* | 0.581(0.470,0.717)* |
| 9 | 0.950(0.897,1.007) | 0.939(0.885,0.996)* | 0.585(0.476,0.720)* |
| 10 | 0.954(0.902,1.009) | 0.943(0.890,0.999)* | 0.596(0.487,0.73)* |
| 11 | 0.958(0.907,1.011) | 0.947(0.895,1.002) | 0.611(0.501,0.745)* |
| 12 | 0.962(0.912,1.014) | 0.951(0.900,1.006) | 0.630(0.518,0.767)* |
| 13 | 0.965(0.914,1.019) | 0.956(0.903,1.011) | 0.651(0.535,0.792)* |
| 14 | 0.968(0.916,1.024) | 0.960(0.905,1.017) | 0.673(0.552,0.821)* |
| 15 | 0.971(0.916,1.029) | 0.963(0.906,1.023) | 0.695(0.568,0.851)* |
| 16 | 0.973(0.915,1.035) | 0.966(0.906,1.029) | 0.717(0.583,0.881)* |
| 17 | 0.974(0.914,1.039) | 0.968(0.905,1.034) | 0.736(0.597,0.907)* |
| 18 | 0.975(0.913,1.041) | 0.969(0.905,1.037) | 0.752(0.609,0.928)* |
| 19 | 0.975(0.912,1.042) | 0.969(0.904,1.038) | 0.764(0.62,0.942)* |
| 20 | 0.974(0.911,1.041) | 0.968(0.904,1.037) | 0.772(0.628,0.949)* |
| 21 | 0.972(0.911,1.038) | 0.966(0.903,1.034) | 0.775(0.633,0.949)* |
| 22 | 0.970(0.910,1.033) | 0.964(0.903,1.029) | 0.773(0.634,0.942)* |
| 23 | 0.967(0.910,1.027) | 0.961(0.902,1.022) | 0.766(0.632,0.929)* |
| 24 | 0.963(0.909,1.021) | 0.957(0.901,1.016) | 0.755(0.624,0.914)* |
| 25 | 0.959(0.907,1.015) | 0.952(0.899,1.009) | 0.741(0.611,0.897)* |
| 26 | 0.955(0.904,1.010) | 0.948(0.896,1.003) | 0.723(0.594,0.88)* |
| 27 | 0.951(0.900,1.006) | 0.944(0.892,0.998)* | 0.704(0.574,0.865)* |
| 28 | 0.948(0.896,1.003) | 0.939(0.887,0.995)* | 0.685(0.552,0.849)* |
| 29 | 0.945(0.892,1.001) | 0.936(0.883,0.992)* | 0.666(0.531,0.835)* |
| 30 | 0.942(0.889,0.999) | 0.933(0.879,0.990)* | 0.649(0.513,0.821)* |
| 31 | 0.941(0.888,0.998) | 0.931(0.878,0.988)* | 0.635(0.498,0.808)* |
| 32 | 0.942(0.889,0.997) | 0.931(0.879,0.987)* | 0.625(0.49,0.797)* |
| 33 | 0.944(0.892,0.999) | 0.933(0.881,0.989)* | 0.621(0.488,0.790)* |
| 34 | 0.948(0.893,1.007) | 0.938(0.882,0.997)* | 0.624(0.491,0.794)* |
| 35 | 0.955(0.888,1.028) | 0.946(0.877,1.019) | 0.637(0.495,0.819)* |
| 36 | 0.966(0.873,1.068) | 0.957(0.864,1.060) | 0.662(0.494,0.886)* |
| 37 | 0.980(0.850,1.131) | 0.972(0.841,1.123) | 0.703(0.484,1.020) |

O_3_ exposure and the risk of PTB in specific gestational weeks. Distribution lag nonlinear model combined with a quasi-poisson regression were applied to estimate aRR (95%CI) of PTB with different percentiles (75^th^, 85^th^, and 95^th^) of O_3_ relative to the 25^th^ percentile (6.0 μg/m^3^); All models were adjusted for the day of week and season; * *P* < 0.05
