# Supplementary material for: The impact of extreme air pollution on preterm birth in twin pregnancies: identifying susceptible exposure windows
Source: Ann Med. 2025 Jul 20;57(1):2534854. doi: 10.1080/07853890.2025.2534854 (PMC12278472; doi:10.1080/07853890.2025.2534854)
Supplement: Supplemental Material [file IANN_A_2534854_SM9594.zip › Supplemental/Table S7.docx]

**Table S7.** Model parameter selection for different degrees of freedom of PM_2.5_, PM_10_, NO_2_, SO_2_, CO, and O_3_

| **Variable** | **Degree of air pollutants** | | | | | |
| --- | --- | --- | --- | --- | --- | --- |
|  | **3** | | **4** | | **5** | |
|  | **QAIC** | **QBIC** | **QAIC** | **QBIC** | **QAIC** | **QBIC** |
| PM_2.5_ | 5645.956 | 5756.456 | 5646.150 | 5767.797 | 5644.307 | 5776.766 |
| PM_10_ | 5646.150 | 5756.642 | 5645.996 | 5767.655 | 5644.443 | 5776.786 |
| NO_2_ | 5651.287 | 5762.435 | 5649.484 | 5771.679 | 5651.136 | 5784.447 |
| SO_2_ | 5643.026 | 5753.161 | 5641.848 | 5762.938 | 5636.437 | 5767.715 |
| CO | 5644.889 | 5755.032 | 5644.005 | 5765.287 | 5645.799 | 5778.086 |
| O_3_ | 5634.759 | 5744.292 | 5634.659 | 5755.023 | 5638.148 | 5769.621 |

Abbreviations: PM_2.5_, particulate matter with an aerodynamic diameter ≤ 2.5μm; PM_10_, particulate matter with an aerodynamic diameter ≤ 10μm; SO_2_, sulfur dioxide; NO_2_, nitrogen dioxide; CO, carbon monoxide; O_3_, ozone.
