# Supplementary material for: The impact of extreme air pollution on preterm birth in twin pregnancies: identifying susceptible exposure windows
Source: Ann Med. 2025 Jul 20;57(1):2534854. doi: 10.1080/07853890.2025.2534854 (PMC12278472; doi:10.1080/07853890.2025.2534854)
Supplement: Supplemental Material [file IANN_A_2534854_SM9594.zip › Supplemental/Table S8.docx]

**Table S8. Associations between extreme pollution events and spontaneous preterm birth.**

| **Variables** | **0-1 lag week** | | **0-1 lag month** | | **0-3 lag months** | | **0-6 lag months** | | **0-9 lag months** | |
| --- | --- | --- | --- | --- | --- | --- | --- | --- | --- | --- |
|  | **aOR(95%CI)** | **p-value** | **aOR(95%CI)** | **p-value** | **aOR(95%CI)** | **p-value** | **aOR(95%CI)** | **p-value** | **aOR(95%CI)** | **p-value** |
| PM_2.5_ |  |  |  |  |  |  |  |  |  |  |
| 90^th^-days | 1.044(0.966,1.126) | 0.267 | 1.043(1.017,1.069) | 0.001* | 1.020(1.010,1.030) | <0.001* | 1.016(1.009,1.023) | <0.001* | 1.018(1.011,1.024) | <0.001* |
| 90^th^-2D | 1.038(0.938,1.143) | 0.459 | 1.068(1.031,1.106) | <0.001* | 1.029(1.015,1.044) | <0.001* | 1.024(1.013,1.034) | <0.001* | 1.025(1.015,1.035) | <0.001* |
| 95^th^-days | 1.044(0.926,1.170) | 0.472 | 1.081(1.039,1.125) | <0.001* | 1.040(1.023,1.058) | <0.001* | 1.034(1.022,1.047) | <0.001* | 1.037(1.025,1.049) | <0.001* |
| 95^th^-2D | 1.057(0.901,1.227) | 0.480 | 1.122(1.060,1.188) | <0.001* | 1.067(1.041,1.094) | <0.001* | 1.060(1.040,1.081) | <0.001* | 1.044(0.966,1.126) | 0.267 |
| PM_10_ |  |  |  |  |  |  |  |  |  |  |
| 90^th^-days | 1.039(0.969,1.112) | 0.278 | 1.038(1.016,1.062) | 0.001* | 1.018(1.009,1.027) | <0.001* | 1.015(1.009,1.022) | <0.001* | 1.017(1.011,1.022) | <0.001* |
| 90^th^-2D | 1.025(0.935,1.120) | 0.586 | 1.053(1.020,1.086) | 0.001* | 1.026(1.013,1.039) | <0.001* | 1.021(1.012,1.031) | <0.001* | 1.024(1.015,1.033) | <0.001* |
| 95^th^-days | 1.066(0.949,1.192) | 0.272 | 1.087(1.044,1.131) | <0.001* | 1.040(1.023,1.058) | <0.001* | 1.034(1.021,1.046) | <0.001* | 1.037(1.025,1.049) | <0.001* |
| 95^th^-2D | 1.092(0.940,1.260) | 0.234 | 1.131(1.070,1.196) | <0.001* | 1.069(1.043,1.095) | <0.001* | 1.061(1.042,1.080) | <0.001* | 1.039(0.969,1.112) | 0.278 |
| SO_2_ |  |  |  |  |  |  |  |  |  |  |
| 90^th^-days | 1.118(1.012,1.234) | 0.027* | 1.071(1.036,1.108) | <0.001* | 1.036(1.022,1.050) | <0.001* | 1.031(1.021,1.041) | <0.001* | 1.034(1.024,1.043) | <0.001* |
| 90^th^-2D | 1.161(0.926,1.443) | 0.183 | 1.107(1.025,1.194) | 0.009* | 1.080(1.045,1.115) | <0.001* | 1.064(1.040,1.089) | <0.001* | 1.067(1.045,1.089) | <0.001* |
| 95^th^-days | 1.226(1.055,1.421) | 0.007* | 1.141(1.076,1.209) | <0.001* | 1.074(1.046,1.102) | <0.001* | 1.064(1.044,1.085) | <0.001* | 1.071(1.051,1.092) | <0.001* |
| 95^th^-2D | 1.349(0.920,1.970) | 0.116 | 1.205(1.018,1.421) | 0.028* | 1.161(1.079,1.247) | <0.001* | 1.118(1.063,1.176) | <0.001* | 1.118(1.012,1.234) | 0.027* |
| CO |  |  |  |  |  |  |  |  |  |  |
| 90^th^-days | 1.012(0.924,1.107) | 0.804 | 1.037(0.997,1.078) | 0.071 | 1.017(0.998,1.035) | 0.079 | 1.017(1.004,1.030) | 0.010* | 1.018(1.007,1.029) | 0.001* |
| 90^th^-2D | 1.026(0.846,1.237) | 0.790 | 1.093(1.002,1.191) | 0.043* | 1.058(1.015,1.102) | 0.007* | 1.053(1.025,1.082) | <0.001* | 1.051(1.029,1.074) | <0.001* |
| 95^th^-days | 1.110(0.987,1.246) | 0.080 | 1.067(1.022,1.114) | 0.003* | 1.030(1.011,1.048) | 0.001* | 1.028(1.015,1.041) | <0.001* | 1.031(1.020,1.042) | <0.001* |
| 95^th^-2D | 0.924(0.644,1.297) | 0.658 | 1.087(0.944,1.249) | 0.241 | 1.040(0.981,1.103) | 0.185 | 1.033(0.998,1.069) | 0.061 | 1.012(0.924,1.107) | 0.804 |
| O_3_ |  |  |  |  |  |  |  |  |  |  |
| 90^th^-days | 1.118(0.998,1.252) | 0.053 | 1.105(1.048,1.165) | <0.001* | 1.066(1.038,1.094) | <0.001* | 1.045(1.028,1.062) | <0.001* | 1.040(1.027,1.053) | <0.001* |
| 90^th^-2D | 1.166(0.855,1.572) | 0.321 | 1.072(0.915,1.251) | 0.385 | 1.055(0.969,1.147) | 0.216 | 1.055(1.002,1.110) | 0.041* | 1.054(1.011,1.099) | 0.013* |
| 95^th^-days | 1.122(0.939,1.337) | 0.201 | 1.057(0.974,1.147) | 0.179 | 1.056(1.007,1.107) | 0.023* | 1.060(1.030,1.090) | <0.001* | 1.051(1.027,1.075) | <0.001* |
| 95^th^-2D | 0.765(0.249,1.962) | 0.604 | 1.086(0.651,1.756) | 0.744 | 0.936(0.696,1.247) | 0.658 | 1.071(0.916,1.247) | 0.383 | 1.118(0.998,1.252) | 0.053 |
| NO_2_ |  |  |  |  |  |  |  |  |  |  |
| 90^th^-days | 0.932(0.829,1.045) | 0.229 | 0.962(0.912,1.014) | 0.148 | 0.951(0.921,0.981) | 0.002* | 0.951(0.928,0.974) | <0.001* | 0.949(0.928,0.970) | <0.001* |
| 90^th^-2D | 0.994(0.690,1.403) | 0.972 | 0.864(0.718,1.032) | 0.112 | 0.928(0.830,1.035) | 0.185 | 0.997(0.917,1.084) | 0.953 | 0.978(0.905,1.056) | 0.563 |
| 95^th^-days | 0.932(0.829,1.045) | 0.229 | 0.962(0.912,1.014) | 0.148 | 0.951(0.921,0.981) | 0.002* | 0.951(0.928,0.974) | <0.001* | 0.949(0.928,0.970) | <0.001* |
| 95^th^-2D | 0.994(0.690,1.403) | 0.972 | 0.864(0.718,1.032) | 0.112 | 0.928(0.83,1.035) | 0.185 | 0.997(0.917,1.084) | 0.953 | 0.978(0.905,1.056) | 0.563 |

Associations between extreme pollution events and spontaneous preterm birth. Multivariate logistic regression models were applied to estimate aOR (95%CI) of PTB. All models were adjusted for age, PBMI, Gravidity, Nulliparity, IVF, DCDA, scarred uterus, placenta previa, FGR, GDM, PE. 90th-days, 90th-2D, 95th-days and 95th-2D represent the frequency of extreme pollution exposure indices. The 90th-days and 95th-days indices represent the total number of days within a specific exposure window where air pollutant concentrations reach or exceed the 90th and 95th percentiles, respectively, while the 90th-2D and 95th-2D indices indicate the frequency of concentrations reaching or exceeding these percentiles on two consecutive days. When lag days are 0, it refers to the time of delivery.

Abbreviations:PM_2.5_, particulate matter with an aerodynamic diameter ≤ 2.5μm; PM_10_, particulate matter with an aerodynamic diameter ≤ 10μm; SO_2_, sulfur dioxide; NO_2_, nitrogen dioxide; CO, carbon monoxide; O_3_, ozone; PBMI, Pre-pregnancy Body Mass Index. ****P* < 0.05.**
