# Supplementary material for: The impact of extreme air pollution on preterm birth in twin pregnancies: identifying susceptible exposure windows
Source: Ann Med. 2025 Jul 20;57(1):2534854. doi: 10.1080/07853890.2025.2534854 (PMC12278472; doi:10.1080/07853890.2025.2534854)
Supplement: Supplemental Material [file IANN_A_2534854_SM9594.zip › Supplemental/Table S9.docx]

**Table S9. Associations between extreme pollution events and iatrogenic preterm birth.**

| **Variables** | **0-1 lag week** | | **0-1 lag month** | | **0-3 lag months** | | **0-6 lag months** | | **0-9 lag months** | |
| --- | --- | --- | --- | --- | --- | --- | --- | --- | --- | --- |
|  | **aOR(95%CI)** | **p-value** | **aOR(95%CI)** | **p-value** | **aOR(95%CI)** | **p-value** | **aOR(95%CI)** | **p-value** | **aOR(95%CI)** | **p-value** |
| PM_2.5_ |  |  |  |  |  |  |  |  |  |  |
| 90^th^-days | 0.980(0.919,1.044) | 0.532 | 1.005(0.983,1.027) | 0.649 | 1.001(0.992,1.010) | 0.825 | 1.000(0.994,1.006) | 0.926 | 1.000(0.995,1.005) | 0.989 |
| 90^th^-2D | 0.970(0.891,1.053) | 0.466 | 1.010(0.979,1.041) | 0.535 | 1.002(0.990,1.014) | 0.778 | 1.000(0.992,1.009) | 0.947 | 1.000(0.992,1.008) | 0.987 |
| 95^th^-days | 0.969(0.876,1.068) | 0.529 | 1.014(0.978,1.052) | 0.441 | 1.004(0.989,1.019) | 0.598 | 1.003(0.993,1.014) | 0.544 | 1.003(0.993,1.012) | 0.591 |
| 95^th^-2D | 0.968(0.845,1.104) | 0.635 | 1.016(0.963,1.071) | 0.565 | 1.009(0.987,1.031) | 0.443 | 1.007(0.991,1.023) | 0.391 | 0.980(0.919,1.044) | 0.532 |
| PM_10_ |  |  |  |  |  |  |  |  |  |  |
| 90^th^-days | 0.985(0.929,1.043) | 0.599 | 1.006(0.987,1.025) | 0.562 | 1.001(0.994,1.009) | 0.713 | 1.001(0.996,1.006) | 0.686 | 1.001(0.996,1.005) | 0.792 |
| 90^th^-2D | 0.973(0.901,1.048) | 0.472 | 1.007(0.980,1.034) | 0.619 | 1.002(0.991,1.012) | 0.755 | 1.001(0.994,1.009) | 0.757 | 1.001(0.994,1.009) | 0.717 |
| 95^th^-days | 0.992(0.899,1.091) | 0.865 | 1.017(0.981,1.054) | 0.352 | 1.004(0.990,1.019) | 0.556 | 1.004(0.994,1.014) | 0.457 | 1.002(0.993,1.012) | 0.611 |
| 95^th^-2D | 0.991(0.870,1.125) | 0.891 | 1.027(0.975,1.081) | 0.315 | 1.010(0.989,1.032) | 0.351 | 1.008(0.993,1.024) | 0.290 | 0.985(0.929,1.043) | 0.599 |
| SO_2_ |  |  |  |  |  |  |  |  |  |  |
| 90^th^-days | 1.028(0.945,1.117) | 0.523 | 1.016(0.988,1.046) | 0.260 | 1.006(0.994,1.018) | 0.314 | 1.004(0.996,1.012) | 0.344 | 1.002(0.995,1.010) | 0.532 |
| 90^th^-2D | 0.988(0.811,1.197) | 0.901 | 0.992(0.926,1.062) | 0.817 | 1.009(0.980,1.039) | 0.533 | 1.011(0.991,1.031) | 0.279 | 1.011(0.994,1.029) | 0.207 |
| 95^th^-days | 1.104(0.973,1.251) | 0.123 | 1.050(0.999,1.102) | 0.053 | 1.015(0.993,1.037) | 0.177 | 1.008(0.992,1.024) | 0.320 | 1.007(0.992,1.022) | 0.382 |
| 95^th^-2D | 1.297(0.922,1.846) | 0.138 | 1.120(0.967,1.298) | 0.13 | 1.043(0.978,1.112) | 0.196 | 1.021(0.977,1.067) | 0.349 | 1.028(0.945,1.117) | 0.523 |
| CO |  |  |  |  |  |  |  |  |  |  |
| 90^th^-days | 0.944(0.878,1.015) | 0.121 | 0.974(0.943,1.005) | 0.094 | 0.990(0.975,1.005) | 0.178 | 0.994(0.984,1.005) | 0.304 | 0.990(0.981,0.999) | 0.028* |
| 90^th^-2D | 0.935(0.802,1.089) | 0.390 | 0.967(0.900,1.039) | 0.36 | 0.987(0.953,1.021) | 0.447 | 1.005(0.982,1.027) | 0.695 | 0.997(0.978,1.016) | 0.735 |
| 95^th^-days | 1.007(0.913,1.109) | 0.892 | 0.999(0.963,1.036) | 0.964 | 1.000(0.984,1.015) | 0.957 | 1.001(0.990,1.011) | 0.915 | 0.998(0.989,1.007) | 0.653 |
| 95^th^-2D | 1.037(0.791,1.353) | 0.791 | 0.968(0.859,1.090) | 0.595 | 0.976(0.928,1.025) | 0.335 | 0.983(0.955,1.012) | 0.257 | 0.944(0.878,1.015) | 0.121 |
| O_3_ |  |  |  |  |  |  |  |  |  |  |
| 90^th^-days | 1.055(0.962,1.157) | 0.256 | 1.019(0.977,1.063) | 0.377 | 0.996(0.976,1.016) | 0.69 | 1.000(0.988,1.013) | 0.951 | 1.000(0.990,1.010) | 0.958 |
| 90^th^-2D | 0.984(0.760,1.271) | 0.903 | 0.977(0.862,1.107) | 0.717 | 0.966(0.902,1.034) | 0.318 | 0.979(0.939,1.021) | 0.324 | 0.978(0.945,1.011) | 0.189 |
| 95^th^-days | 1.079(0.934,1.245) | 0.301 | 0.974(0.911,1.041) | 0.439 | 0.982(0.946,1.020) | 0.347 | 0.996(0.972,1.020) | 0.736 | 0.996(0.977,1.015) | 0.681 |
| 95^th^-2D | 0.856(0.375,1.853) | 0.698 | 0.841(0.540,1.288) | 0.432 | 0.864(0.682,1.092) | 0.224 | 0.956(0.838,1.089) | 0.502 | 1.055(0.962,1.157) | 0.256 |
| NO_2_ |  |  |  |  |  |  |  |  |  |  |
| 90^th^-days | 0.910(0.829,0.999) | 0.048* | 0.948(0.908,0.989) | 0.015* | 0.970(0.947,0.995) | 0.017* | 0.979(0.960,0.998) | 0.030* | 0.971(0.954,0.988) | 0.001* |
| 90^th^-2D | 0.757(0.558,1.019) | 0.070 | 0.864(0.748,0.995) | 0.044* | 0.948(0.870,1.033) | 0.224 | 1.010(0.946,1.078) | 0.766 | 0.977(0.920,1.037) | 0.448 |
| 95^th^-days | 0.910(0.829,0.999) | 0.048* | 0.948(0.908,0.989) | 0.015* | 0.97(0.947,0.995) | 0.017* | 0.979(0.96,0.998) | 0.030* | 0.971(0.954,0.988) | 0.001* |
| 95^th^-2D | 0.757(0.558,1.019) | 0.070 | 0.864(0.748,0.995) | 0.044* | 0.948(0.87,1.033) | 0.224 | 1.01(0.946,1.078) | 0.766 | 0.977(0.920,1.037) | 0.448 |

Associations between extreme pollution events and iatrogenic preterm birth. Multivariate logistic regression models were applied to estimate aOR (95%CI) of PTB. All models were adjusted for age, PBMI, Gravidity, Nulliparity, IVF, DCDA, scarred uterus, placenta previa, FGR, GDM, PE. 90th-days, 90th-2D, 95th-days and 95th-2D represent the frequency of extreme pollution exposure indices. The 90th-days and 95th-days indices represent the total number of days within a specific exposure window where air pollutant concentrations reach or exceed the 90th and 95th percentiles, respectively, while the 90th-2D and 95th-2D indices indicate the frequency of concentrations reaching or exceeding these percentiles on two consecutive days. When lag days are 0, it refers to the time of delivery.

Abbreviations:PM_2.5_, particulate matter with an aerodynamic diameter ≤ 2.5μm; PM_10_, particulate matter with an aerodynamic diameter ≤ 10μm; SO_2_, sulfur dioxide; NO_2_, nitrogen dioxide; CO, carbon monoxide; O_3_, ozone; PBMI, Pre-pregnancy Body Mass Index. ****P* < 0.05.**
